# Base editing rescue of spinal muscular atrophy in cells and in mice

**DOI:** 10.1126/science.adg6518

**Published:** 2023-04-14

**Authors:** Mandana Arbab, Zaneta Matuszek, Kaitlyn M. Kray, Ailing Du, Gregory A. Newby, Anton J. Blatnik, Aditya Raguram, Michelle F. Richter, Kevin T. Zhao, Jonathan M. Levy, Max W. Shen, W. David Arnold, Dan Wang, Jun Xie, Guangping Gao, Arthur H. M. Burghes, David R. Liu

**Affiliations:** 1 Department of Neurology, Rosamund Stone Zander Translational Neuroscience Center, Boston Children's Hospital, Boston, MA 02115, USA.; 2 Department of Neurobiology, Harvard Medical School, Boston, MA 02115, USA.; 3 Merkin Institute of Transformative Technologies in Healthcare, Broad Institute of Harvard and MIT, Cambridge, MA 02142, USA.; 4 Department of Chemistry and Chemical Biology, Harvard University, Cambridge, MA 02138, USA.; 5 Department of Molecular and Cellular Biology, Harvard University, Cambridge, MA, 02138, USA.; 6 Department of Biological Chemistry and Pharmacology, The Ohio State University Wexner Medical Center, 1060 Carmack Road, Columbus, OH 43210, USA.; 7 Horae Gene Therapy Center, University of Massachusetts, Medical School, Worcester, MA 01605, USA.; 8 Computational and Systems Biology Program, Massachusetts Institute of Technology, Cambridge, MA 02139, USA.; 9 Department of Neurology, The Ohio State University Wexner Medical Center, 1060 Carmack Road, Columbus, OH 43210, USA.; 10 NextGen Precision Health, University of Missouri, Columbia, MO 65212, USA.; 11 Horae Gene Therapy Center and RNA Therapeutics Institute, University of Massachusetts, Medical School, Worcester, MA 01605, USA.; 12 Microbiology and Physiological Systems, University of Massachusetts, Medical School, Worcester, MA 01605, USA.; 13 Howard Hughes Medical Institute, Harvard University, Cambridge, MA 02138, USA.

## Abstract

Spinal muscular atrophy (SMA), the leading genetic cause of infant mortality, arises from SMN protein insufficiency following *SMN1* loss. Approved therapies circumvent endogenous SMN regulation and require repeated dosing or may wane. We describe genome editing of *SMN2*, an insufficient copy of *SMN1* harboring a C6>T mutation, to permanently restore SMN protein levels and rescue SMA phenotypes. We used nucleases or base editors to modify five *SMN2* regulatory regions. Base editing converted *SMN2* T6>C, restoring SMN protein levels to wild-type. AAV9-mediated base editor delivery in Δ7SMA mice yielded 87% average T6>C conversion, improved motor function, and extended average lifespan, which was enhanced by one-time base editor+nusinersen co-administration (111 versus 17 days untreated). These findings demonstrate the potential of a one-time base editing treatment for SMA.

## One-Sentence Summary

Base editing enables a one-time treatment for spinal muscular atrophy (SMA) that rescues disease pathology and extends lifespan in mice.

**Fig. 1.**
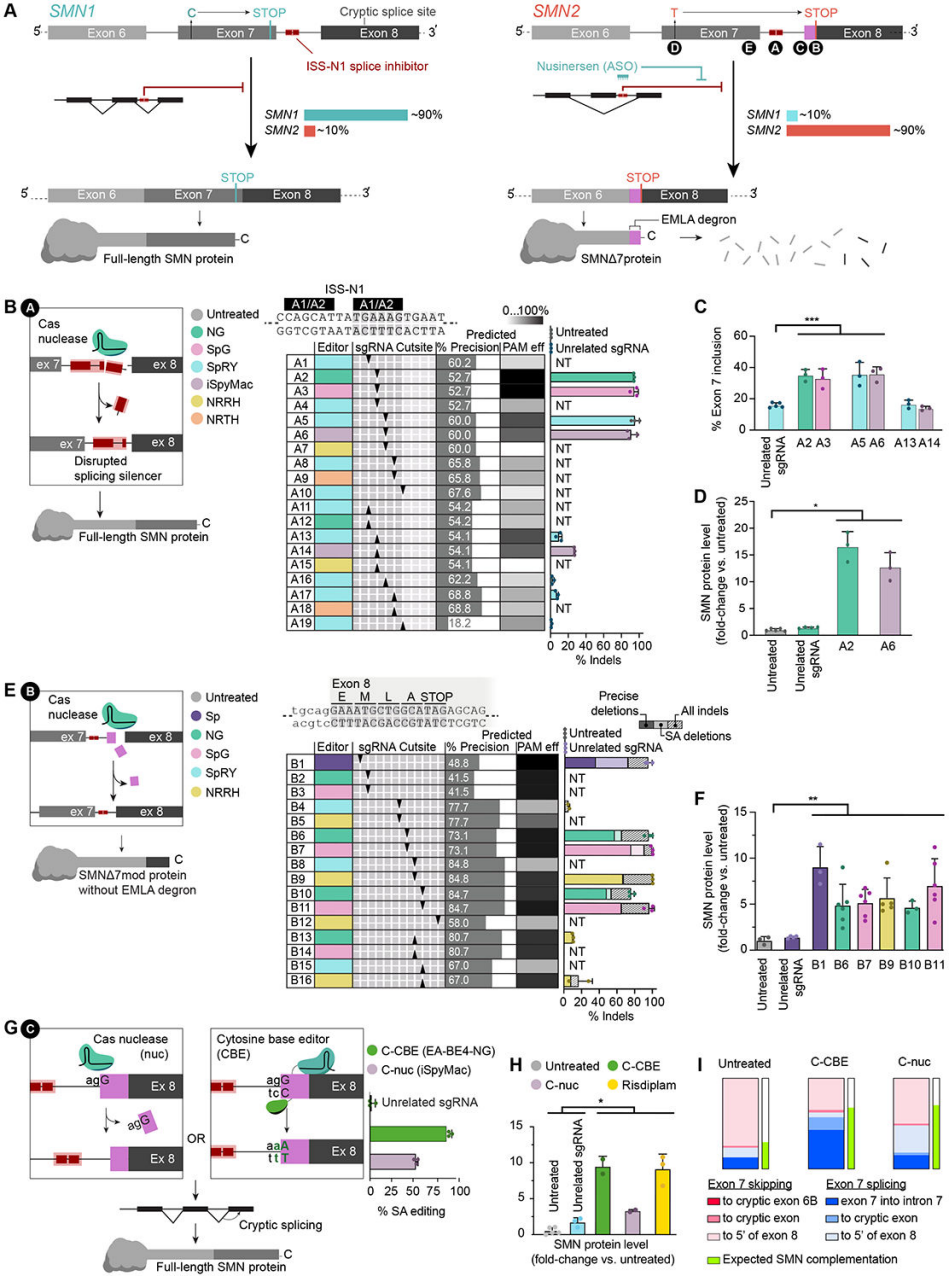
Editing *SMN2* regulatory regions. **(A)** Genomic *SMN* exons 6 to 8, and *SMN* mRNA and protein products. **(B)** Nuclease editing strategy and genome editing outcomes of ISS-N1 targeting (strategy A). The table shows combinations of six nucleases, paired with ten sgRNAs complementary to the top (A1-10) or bottom strand (A11-19) identified by arrows that show the DSB site of the sgRNAs relative to the sequence above. **(C)** Exon 7 inclusion in *SMN* mRNA after editing, as indicated, measured by automated electrophoresis. **(D)** SMN protein levels after editing, as indicated, normalized to histone H3. **(E)** Nuclease editing strategy targeting and genome editing outcomes of targeting the first five codons of exon 8 (strategy B). The table shows combinations of five nucleases, paired with nine sgRNAs complementary to the top (B1-12) or bottom strand (B13-16) identified by arrows that indicate their DSB site, as above. **(F)** Total SMN protein levels after editing. **(G)** Nuclease and cytosine base editing strategies and genome editing outcomes of 3’-splice acceptor disruption at exon 8 (strategy C). (**H)** SMN protein levels following C-nuc and C-CBE editing or treatment with risdiplam, normalized to histone H3. **i)** Distribution of *SMN2* transcript variants after C-nuc and C-CBE editing. Experiments are performed in Δ7SMA mESCs, NT=no treatment, *≤0.05, **≤0.01, ***≤0.005.

SMA is a progressive motor neuron disease and the leading genetic cause of infant mortality(1-3). SMA is caused by homozygous loss or mutation of the essential survival motor neuron 1 *(SMN1)* gene. One or more copies of the nearly identical (>99.9% sequence identity) *SMN2* partially compensates for the loss of *SMN1*(1, 4, 5). However, *SMN1* and *SMN2* differ by a silent C•G-to-T•A substitution at nucleotide position 6 of exon 7 (C6T) that results in exon 7 skipping in mRNA transcripts (Fig. 1A)(6, 7). The resulting truncated SMNΔ7 protein is rapidly degraded, causing SMN protein insufficiency that results in loss of motor neurons, paralysis, and death(8-10). Untreated patients with the most common form of SMA (type I) live a median of 6 months(11, 12).

Upregulation of SMN protein can rescue motor function and substantially improve the prognosis of SMA patients(13-15). However, endogenous SMN protein is subject to multiple levels of regulation that differs across tissues(16-18). While *SMN* underexpression can fail to rescue SMN phenotypes, *SMN* overexpression can cause aggregation, toxicity, and tissue pathology(19-21). Three breakthrough therapeutics effectively rescue many SMA phenotypes and improve lifespan by upregulating SMN protein(22). The antisense oligonucleotide (ASO) nusinersen (Spinraza) and the small-molecule risdiplam (Evrysdi) both promote splicing inclusion of exon 7 resulting in ~2-fold upregulation of SMN levels, and have proven highly effective in the clinic(23, 24). However, SMN protein is reduced ~6.5-fold in the spinal cord of untreated SMA patients(25-27). The partial recovery of SMN protein promoted by these therapeutics may be insufficient at early timepoints and in damaged tissues, potentially underlying the limited rescue observed in some patients(28, 29). Moreover, the transient nature of these therapeutics necessitates repeated administration of costly drugs throughout patients’ lifetimes(30, 31).

AAV-mediated gene complementation of full-length SMN cDNA by onasemnogene abeparvovec-xioi (Zolgensma) leads to constitutive production of SMN in transduced cells that is not under endogenous control(32-34). In the spinal cord, Zolgensma upregulates SMN transcript levels by ~25%(35), while in other tissues such as the liver and dorsal root ganglia, gene complementation may cause *SMN* overexpression that under some circumstances can cause long-term toxicity(21). We do not yet know whether *SMN* overexpression induces toxicity in patients treated with Zolgensma, nor how long AAV-mediated expression will persist in motor neurons in patients (36, 37). As such, a therapeutic modality that restores endogenous gene expression and preserves native SMN regulation by a one-time permanent treatment may address remaining limitations of existing SMA therapies. Genome editing of *SMN2*, which is present in all SMA patients regardless of the nature of their *SMN1* mutation, could enable a one-time treatment for SMA that restores native SMN transcript and protein levels while preserving their endogenous regulatory mechanisms.

## Results

### Predictable and precise nuclease editing of SMN2 ISS-N1 increases SMN protein levels

SMN protein production from *SMN1* and *SMN2* genes is constrained by transcriptional, transcriptomic, and post-translational regulatory sequences. We explored using Cas nucleases to create gain-of-function alleles in *SMN2* regulatory sequences that upregulate SMN levels. The inclusion of exon 7, which underlies SMN protein stability, is strongly influenced by the downstream intronic splicing silencer ISS-N1 that harbors two heterogeneous nuclear ribonucleoprotein (hnRNPs) A1/A2 binding sites (Fig. 1A)(38). Deletions within, and downstream of the 3’ (3-prime) hnRNP A1/A2 binding domain improve exon 7 inclusion(38-41). We speculated that Cas9 nuclease-mediated disruption of the ISS-N1 genomic locus might increase exon 7 inclusion in *SMN2* splicing and thereby increase SMN protein levels (strategy A, Fig. 1B).

We used inDelphi, a machine learning model of SpCas9 nuclease editing outcomes, to predict indel outcomes at the ISS-N1 locus that disrupt hnRNP A1/A2 binding and improve full-length SMN splicing of *SMN2* (Fig. 1B)(42). InDelphi identified ten spacer sequences predicted to induce ≥4-nt deletions at ISS-N1 and loss of ≥1-nt of the 3’-hnRNP A1/A2 domain (‘predicted % precision’). We estimated editing efficiencies of these strategies based on the reported PAM compatibility of these spacer sequences with SpCas9-variant nucleases (‘predicted % PAM efficiency’)(43-46). From 19 possible nuclease editing strategies (A1-A19, defined as different combinations of genome editing agents and guide RNAs) we selected nine (A2, A3, A5, A6, A13, A14, A16, A17, A19) for experimental testing.

We co-transfected Δ7SMA mESCs—which lack endogenous *Smn1,* are homozygous for the full-length human *SMN2* gene, carry human *SMNΔ7*-cDNA transgenes, and harbor a *Mnx1:GFP* reporter of motor neurons (*SMN2*^+/+^*; SMNΔ7; Smn*^−/−^*; Mnx1:GFP)*(47)—with nuclease expression plasmids that carry a blasticidin-resistance cassette and sgRNA plasmids that carry a hygromycin-resistance cassette. Both plasmids also contain Tol2 transposase sequences to enable stable transposon-mediated genomic integration and antibiotic selection. We achieved 92±5.6% average indel frequencies for the top four strategies targeting the ISS-N1 locus (A2, A3, A5, A6, Fig. 1B).

To assess whether nuclease-mediated editing of ISS-N1 improved exon 7 inclusion, we performed reverse-transcription PCR (RT-PCR) of *SMN2* from exons 6 to 8, and quantified *SMNΔ7* and full-length *SMN* products (Fig. 1C). We found that all strategies that edited ISS-N1 with high efficiency (≥85%) resulted in a significant increase in exon 7 inclusion averaging 2.2-fold relative to an unrelated sgRNA control (Welch’s two-tailed t-test *p*=0.01). The increase in exon 7 inclusion caused a substantial increase in SMN protein of 17-fold by A2 and 13-fold by A6 relative to untreated controls (values normalized to histone H3, Welch’s two-tailed t-test *p*=0.02, Fig. 1D, and fig. S1A). Collectively, these results demonstrate that disruption of the ISS-N1 genomic locus can stably increase full-length SMN splicing and protein phenotypes of SMA.

### Predictable and precise genome editing of SMN2 exon 8 increases SMN protein levels

As an alternative nuclease-mediated approach to upregulate SMN levels, we disrupted post-translational regulatory sequences in *SMN2* to increase SMNΔ7 protein stability. The critical difference between full-length SMN and the unstable SMNΔ7 protein is the substitution of 16 amino acids encoded by exon 7 with EMLA, a four-residue degron encoded by exon 8 (Fig. 1A)(8). Extending the coding sequence of exon 8 with five or more heterologous amino acids obscures SMNΔ7 C-terminal degradation signals. These modified SMNΔ7 (SMNΔ7mod) protein variants have increased stability and rescue survival and motor phenotypes of severe SMA mice(48). We designed strategies for Cas nuclease-mediated disruption of exon 8 to generate similar stabilized SMNΔ7mod proteins with therapeutic potential (strategy B1-B16, Supplementary Text, Fig. 1E), and observed up to 7.0-fold increase in SMN protein levels by B11 (Welch’s two-tailed t-test *p*=0.007, Fig. 1F, and fig. S1B).

Some exon 8 editing strategies improved SMN protein stability more than expected based on observed edited genotypes (Fig. 1, E and F). For example, precision edited genotypes were 1.9-fold higher in frequency following B9 editing than B1, yet SMNΔ7mod protein levels were greater in cells edited with B1 (9.1-fold) than B9 (5.7-fold). These data suggest that additional edited genotypes may improve SMN protein stability. Inspection of the non-precisely edited fraction of edited alleles revealed that B1 editing frequently induces indels at the exon 8 splice acceptor. Thus, we hypothesized that disrupting splicing of exon 8 improves SMN protein stability(49).

To test this hypothesis, we disrupted the canonical AG splice acceptor (SA) motif of exon 8 using either a nuclease or cytosine base editor (C-nuc or C-CBE) in Δ7SMA mESCs (Fig. 1G)(45, 50), and observed 54±2.3% indels from C-nuc and 89±2.3% cytosine base editing from C-CBE. Notably, C-nuc editing resulted in a complex mixture of indel genotypes at the intron-exon junction that resulted in deletion of additional nucleotides beyond the AG motif. Both strategies significantly increased SMN levels in Δ7SMA mESCs, similar to treatment with risdiplam (3.3-fold for C-nuc, 9.5-fold for C-CBE, 9.1-fold for risdiplam relative to untreated, Welch’s two-tailed t-test *p*<0.05, Fig. 1H, and fig. S1D to G), indicating that alternative splicing at exon 8 improves the stability of *SMN2* gene products.

We investigated how exon 8 SA disruption affects *SMN2* transcripts (Supplementary Text). C-CBE editing induced a minor increase in *SMN2* mRNA that only partially explains the 9.5-fold increase in SMN levels (fig. S1H). We also observed a profound shift in *SMN2* splice products (Fig. 1I). We investigated whether these alternative splice isoforms improve stability of SMN proteins, and found that transcripts including exon 7 were increased 2-fold by C-CBE (63±2.0%) and 1.6-fold by C-nuc (50±1.1%) relative to untreated cells (24±1.4%). These transcripts often retain intron 7 as in some functional transcript variants of *SMN2* (ENST00000511812.5, fig. S1I). Importantly, all transcripts that include exon 7 encode full-length SMN protein and can therefore complement loss of *SMN1*, Thus, the substantial increase in SMN protein levels following exon 8 SA editing predominantly arises from an increase in normal full-length SMN.

Collectively, the tested *SMN2* editing strategies permanently increase SMN protein levels up to 17-fold (strategy A2), 9.1-fold (strategy B1), and 9.5-fold (strategy C-CBE). As a 1.5- to 2-fold increase in SMN protein is therapeutic for SMA patients(23, 24), these strategies represent promising approaches for further studies.

### Efficient and precise base editing of SMN2 splice regulatory elements

**Fig. 2.**
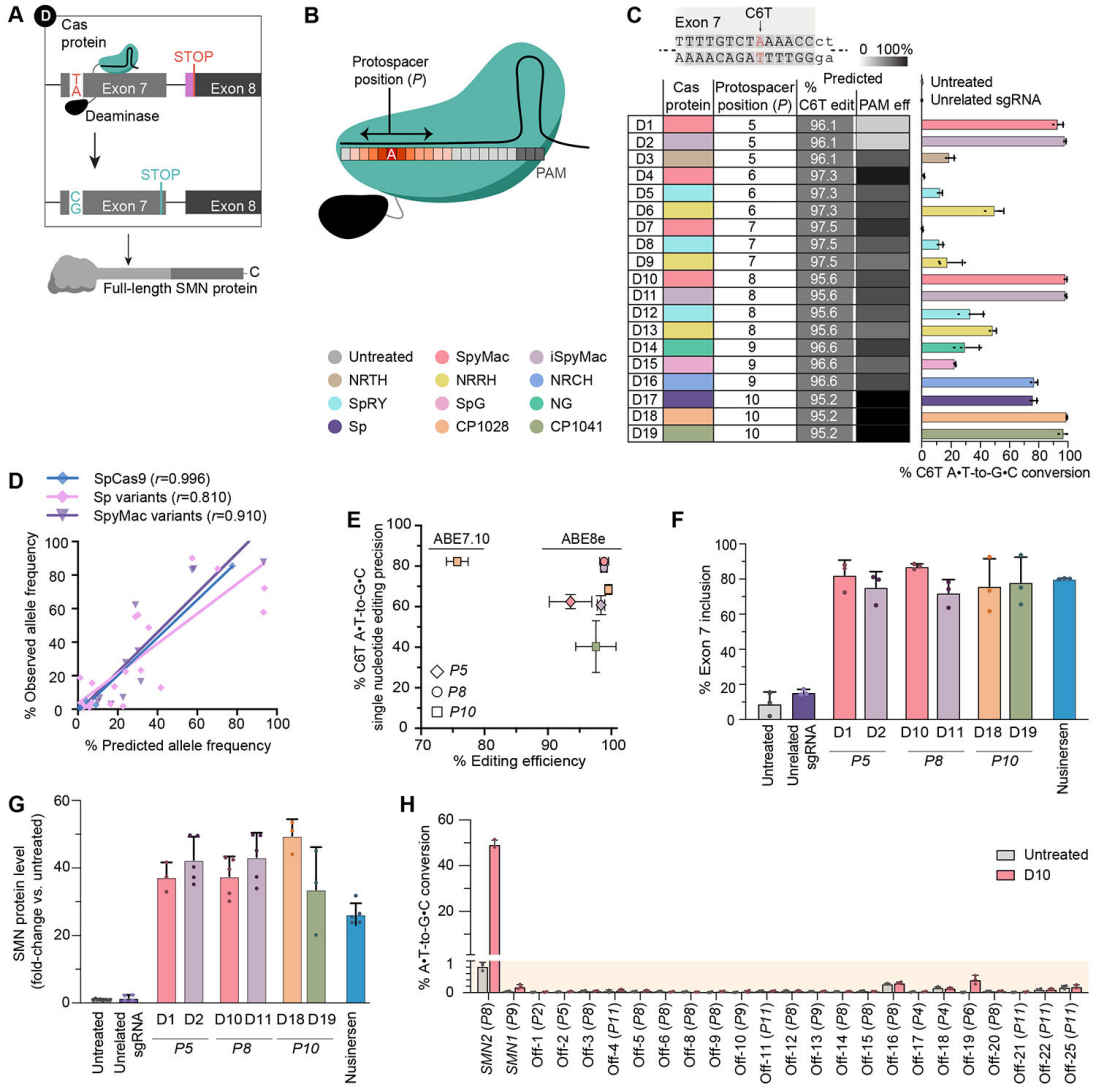
Adenine base editing of *SMN2* C6T. **(A)** Adenine base editing of *SMN2* C6T (strategy D). **(B)** Target nucleotide position within the protospacer (*P#*) for base editing. A typical base editor activity window is illustrated as a heat map. **(C)** The table shows ABE8e editing strategies with color-coded Cas-variant domains and their corresponding spacers. The protospacer position of the C6T target nucleotide (*P#*) is indicated. Graph shows genome editing outcomes in Δ7SMA mESCs. **(D)** Correlation of BE-Hive predicted editing outcomes with observed allele frequencies after base editing with ABE7.10 or ABE8e deaminases fused to different Cas variants. Pearson’s *r* is shown, 95% CI ranges are 0.9408–0.9998 for SpCas9, 0.5823–0.9201 for SpCas9 engineered and evolved variants, and 0.7557–0.9689 for SpyMac Cas variants. **(E)** Plot of base editing efficiency and single nucleotide editing precision of C6T by the indicated ABE and spacer combinations. **(F)** Exon 7 inclusion in *SMN* mRNA after editing by the indicated strategies, measured by automated electrophoresis. **(G)** SMN protein levels after editing by the indicated strategies, normalized to histone H3. **(H)** On-target and off-target base editing of strategy D10 in HEK293T cells. Bars show editing of the most frequently edited nucleotide at each locus, with the *P*# position shown in parenthesis.

Several single-nucleotide substitutions in exon 7 strongly regulate splicing of *SMN2*, including the C-to-T transition at position 6 (C6T) that differentiates *SMN1* (C) from *SMN2* (T) genes (Fig. 1A), and T44C, G52A, and A54G at the 3’-end of exon 7(51). Using existing and newly developed BE-Hive predictive models of base-editing outcomes (Supplementary Text, fig. S2, A to E), we identified 42 strategies (combinations of base editors and guide RNAs) to modify exon 7 splicing regulatory elements (SREs, Fig. 2, A to C and fig. S2, F and G). We designed 13 spacers targeting C6T using ABE8e (strategy D1-19), or targeting C6T, T44C, G52A, and A54G using ABE8e, ABE7.10 and EA-BE4 deaminases (strategy E1-23). We paired these spacers with 12 compatible SpCas9-variants based on reported PAM preferences (‘predicted % PAM efficiency’)(43, 50, 52). We validated these strategies in Δ7SMA mESCs, and found that the BE-Hive models of SpCas9 base editors predicted edited outcomes of Cas-variant base editors with high accuracy (Cas9-NG(52), NRTH, NRRH, NRCH(44) Pearson’s *r*=0.810, chimeric SpyMac and iSpMac(45) Pearson’s *r*=0.910, Supplementary Text, Fig. 2D).

Base editing of exon 7 SREs was highly efficient. At 3’-SREs, we achieved 69±5.0% T44C editing by E14, 92±4.0% G52A editing by E20, and 95±5.1% A54G editing by E23 (fig. S2, F and G). We achieved nearly complete (94%–99.5%) C6T A•T-to-G•C conversion by strategies targeting C6T at positions *P5* (D1, D2), *P8* (D10, D11), and *P10* (D18, D19) within the protospacer (Fig. 2, A to C). The deaminase in ABE7.10 enabled up to 64±2.5% conversion of T6>C (E7, fig. S2G)(53, 54).

The frequency of edited alleles with single-nucleotide T6>C conversion alone (i.e., without any bystander edits or indels) varied substantially between the most efficient C6T editing strategies, ranging from 82±1.9% from D10 to 40±13% from D19 editing (Fig. 2E). Prior studies suggest that the coding sequence at the SMN C-terminus beyond exon 6 does not strongly affect SMN protein function and it is therefore unlikely that single-nucleotide editing precision of C6T is imperative for rescue of SMA(8, 48, 55). Maximizing the sequence similarity of modified *SMN2* genes to native *SMN1*, however, may preserve additional regulatory interactions, including those not yet known. D10, the strategy with the highest precision and efficiency (99±0.7%), did not induce measurable indels and its induced bystander missense nucleotide substitutions (18±2.4%) have previously been shown to benefit inclusion of exon 7 by improved protein binding at the exonic splicing enhancer (fig. S2H)(56, 57). Together, these results establish efficient base editing strategies to convert *SMN2* T6>C with high-fidelity and few undesired byproducts.

### Base editing of SMN2 splice regulatory elements rescues SMN protein levels

Next, we asked whether base editing of exon 7 SREs results in functional rescue of cellular SMA phenotypes. The top six ABE8e editing strategies that converted C6T in >97% of alleles increased exon 7 inclusion to 78±10.2% on average, up to 9.7-fold higher than untreated cells (87±1.5% by D10 compared to 9.0±6.6% in untreated, Welch’s two-tailed t-test *p*<0.002, Fig. 2F). These results are on par with, or exceed, maximum exon 7 inclusion by risdiplam or nusinersen treatment of Δ7SMA mESCs (89±4.3% and 80±0.3%, respectively, Fig. 2F and fig. S1E), and resemble splicing ratios of *SMN1* genes (82±7.3% in U2OS cells)(38, 39). Base editing of 3’-SREs in exon 7 also improved inclusion, averaging 60±3.2% following T44C editing by E14, 76±12% following G52A editing by E20, and 50±8.6% following A54G editing by E23 (fig. S2I). These data demonstrate that base editing of various exon 7 SREs can increase full-length *SMN* splice products.

Base editing of 3’-SREs increased SMN protein levels in ways that did not closely mirror observed improvements in exon 7 inclusion. We detected a 3.4-fold increase in SMN protein by E14 base editing of T44C, 23-fold increase by E20 editing of G52A, and 1.6-fold increase by E23 editing of A54G (Welch’s two-tailed t-test *p*=0.02), despite all three edits inducing comparable improvements in exon 7 inclusion (figs. S2I, and S3, A and B). We hypothesized that unintended bystander edits may underlie this persistent protein instability and found that the T44C and A54G editing strategies frequently ablate the nearby TAA stop codon in exon 7 (fig. S2, F and G). A failure to terminate translation in exon 7 leads to the extension of full-length SMN proteins with the EMLA degron encoded by exon 8 (Fig. 1A). Thus, imprecise editing of T44C or A54G by E14 or E23 results in the translation of unstable full-length SMN-EMLA fusions that prevent upregulation of SMN protein levels. Editing of G52A by E20 uses the EA-BE4 cytosine deaminase that does not recognize TAA as a substrate and therefore does not induce non-silent bystander changes in 99±0.1% of edited alleles, resulting in a 23-fold improvement in SMN protein levels.

Base editing of exon 7 C6T resulted in the greatest upregulation of SMN protein. The top six ABE8e editing strategies that correct C6T in >97% of alleles induced a 41-fold average increase in SMN protein levels compared to untreated controls (normalized to H3, Welch’s two-tailed t-test *p*<0.0002, Fig. 2G and fig. S3C), indicating complete rescue of normal SMN protein levels in Δ7SMA mESCs, which are ~40-fold reduced relative to wild-type mESCs(47). Despite inducing comparable increase in exon 7 inclusion, base editing of C6T enabled a 4.5-fold and 1.5-fold greater increase in SMN protein levels than risdiplam and nusinersen treatment of Δ7SMA mESCs (9-fold and 17-fold respectively, compared to 41-fold on average across the top six strategy D approaches, Fig. 1H, 2G, and figs. S1, D and F to G, and S3, C and E to F). Normal levels of SMN protein are essential to the function, survival, and long-term health of all species in the animal kingdom(58-61). Restoring wild-type levels of SMN protein as achieved by base editing strategy may thus best maximize the long-term health of SMA patients.

Among all genome editing strategies tested, base editing of C6T by D10 induces the greatest increase in exon 7 inclusion (87±1.5%) and best recapitulates native SMN protein levels (95% of wild-type levels, a 38-fold increased versus untreated Δ7SMA mESCs). D10 base editing is highly efficient (99±0.7%) with high on-target precision (82±0.0%). The *SMN2* gene arose from a duplication of the chromosomal region containing *SMN1*, and shares an identical promoter and >99.9% sequence identity with *SMN1*, including 100% DNA conservation of its protein-coding sequence other than exon 7 C6T(1, 4, 5). We performed RT-qPCR and quantified *SMN2* mRNA levels in edited cells, confirming that *SMN2* mRNA abundance is not affected by D10 base editing compared to untreated Δ7SMA mESCs or following ABE8e transfection with an unrelated sgRNA (fig. S3G). Together, these data indicate that D10 editing of *SMN2* faithfully reproduces the genomic sequence and function of native *SMN1* alleles. Therefore, we selected strategy D10 for further study.

### Off-target editing analysis of ABE8e targeting SMN2 C6T in the human genome

Some base editors can induce off-target deamination in cells, including Cas-dependent off-target DNA editing and Cas-independent off-target DNA or RNA editing(62-66). Genomic and transcriptomic off-target deamination by adenine base editors without involvement of the Cas protein component is rare, and deaminase variants that minimize these events have been reported(62, 67). We assessed the Cas-dependent genome specificity of the D10 strategy (ABE8e-SpyMac and *P8* sgRNA) characterizing SpyMac Cas9 nuclease with P8 sgRNA using CIRCLE-seq(68), an unbiased and sensitive empirical *in vitro* off-target detection method. Potential off-target sites nominated by CIRCLE-seq can then be sequenced in-depth in base-edited human cells to provide a sensitive genome-wide analysis of off-target genome editing events induced by the D10 strategy(68, 69).

We generated purified D10 strategy ribonucleoprotein (RNP) complexes containing SpyMac nuclease and *P8* sgRNA to treat human genomic DNA from HEK293T cells *in vitro* and analyzed rare off-target genomic cleavage events (fig. S3H). We identified 55 candidate SpyMac-dependent DNA off-target loci nominated by the CIRCLE-seq method. Next, we measured on-target and genomic off-target editing at the top 23 CIRCLE-seq-nominated loci in human cells (Supplementary Text, Fig. 2H and fig. S3I). We achieved 49±1.8% C6T on-target base editing at *SMN2* in HEK293T cells and observed minimal base editing at *SMN1* (0.15±0.07%), which is generally absent in SMA patients. We detected minor levels of D10 base editing at off-target site ranked 19 (0.41±0.14%), which is in an intergenic region of chromosome 15, and no evident base editing (≤0.03% over untreated cells) at the other 21 assayed potential off-target loci. These data indicate high genomic target specificity of the D10 base editing strategy for the on-target locus.

Together, these experiments did not detect any coding mutations or sequence changes of anticipated physiological significance in the human genome and support continued preclinical evaluation of the D10 strategy, including assessment of base editor off-target editing measured in various tissues that may accumulate over an extended period of time. We refer to the D10 editing strategy as the ‘ABE strategy’ hereafter.

### Viral delivery of ABE enables efficient in vivo conversion of SMN2 C6T

**Fig. 3.**
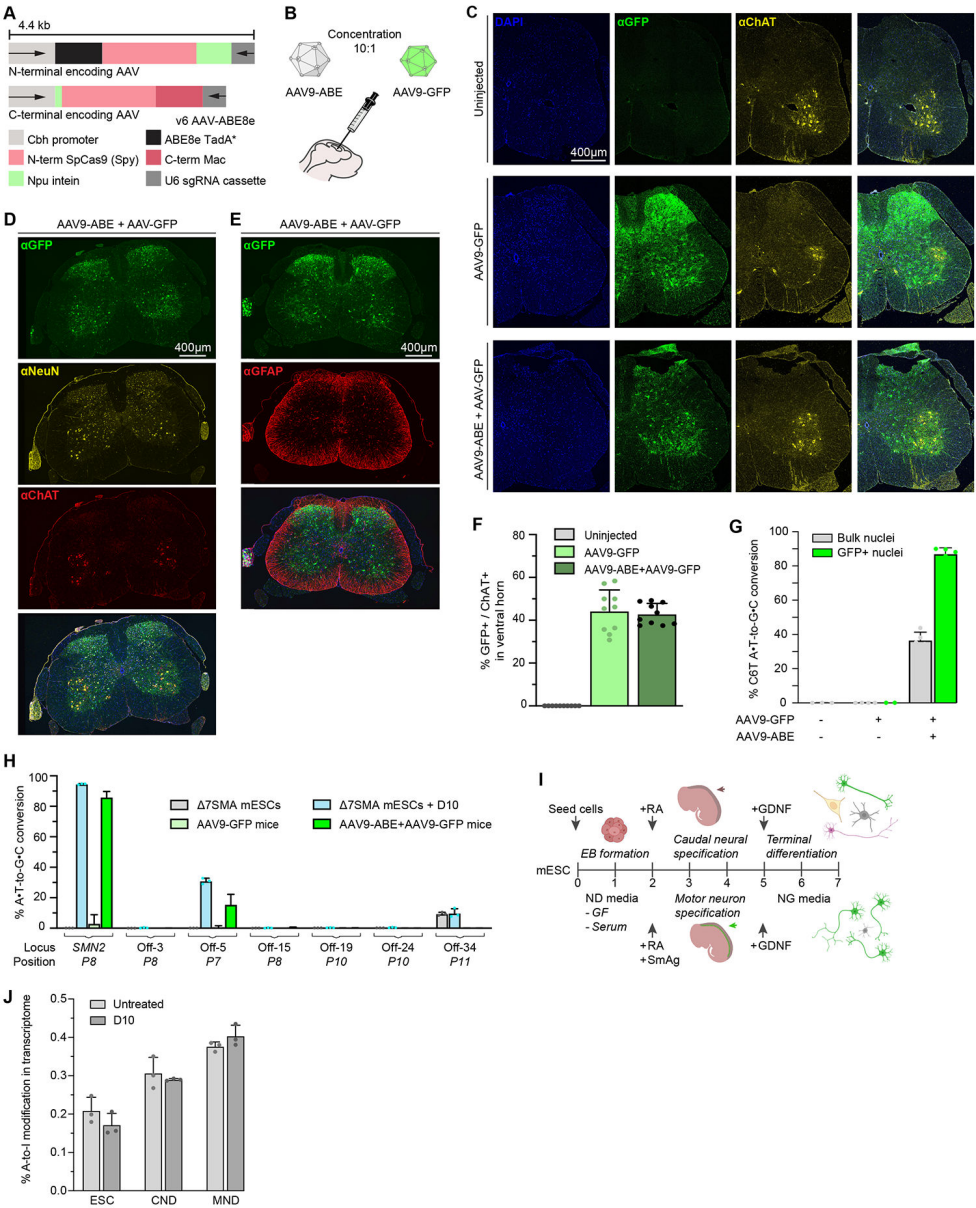
Adenine base editing in Δ7SMA mice. **(A)** Dual-AAV vectors encoding split-intein ABE8e-SpyMac and *P8* sgRNA cassettes, v6 AAV9-ABE8e. **(B)** Neonatal ICV injections in Δ7SMA mice with AAV9-ABE, and AAV9-GFP as a transduction control. **(C-E)** Immunofluorescence images of lumbar spinal cord sections from wild-type Δ7SMA mice at 25 weeks old, ICV injected on PND0-1 with AAV9-ABE, AAV9-GFP, or uninjected as indicated. GFP indicates transduction, ChAT labels spinal motor neurons in the ventral horn, NeuN labels post-mitotic neurons, GFAP labels astrocytes, DAPI stains all nuclei. **(F)** Quantification of GFP and ChAT double-positive cells within the ventral horn (*n*=3). **(G)** Base editing in bulk and GFP+ flow-sorted nuclei of Δ7SMA mice treated with AAV9-ABE+AAV9-GFP (*n*=5), AAV9-GFP (*n*=4), or uninjected (*n*=3). **(H)** On-target and off-target editing following VIVO analysis of strategy D10 in Δ7SMA mESCs compared to AAV9-ABE+AAV9-GFP treatment in Δ7SMA mice. Bars show editing of the most frequently edited nucleotide at each locus, with the *P*# position shown in parenthesis. **(I)** Schematic of motor neuron differentiation (MND) and caudal-neural differentiation (CND) of Δ7SMA mESCs. **(J)** Whole transcriptome A-to-I RNA off-target editing analysis in Δ7SMAmESCs (*n*=3), and CND (*n*=3) and MND (*n*=3) differentiated cells stably expressing the D10 strategy.

To enable *in vivo SMN2* C6T conversion in an animal model of SMA, we designed an adeno-associated virus (AAV) strategy to package ABE8e-SpyMac and the *P8* sgRNA for delivery (v6 AAV-ABE8e, Supplementary Text, Fig. 3A and fig. S3J). The AAV serotype 9 (AAV9) has a well-established tropism for neurons in the CNS of a wide range of organisms, including Δ7SMA mice and human patients(70-72). In the cortex, AAV9 has been shown to almost exclusively target neurons(72), and intracerebroventricular (ICV) or systemic injection in neonates results in efficient transduction of spinal motor neurons to enable rescue of SMA disease phenotypes and lethality in both mice and humans(13, 32, 73). Thus, we selected AAV9 for delivery of our D10 ABE strategy (‘AAV9-ABE’) to Δ7SMA neonates by ICV injection to correct the *SMN2* C6T target *in vivo* (Fig. 3B).

We ICV injected SMA neonates with total 2.7x10^13^ vg/kg of the dual AAV9-ABE vectors, along with 2.7x10^12^ vg/kg AAV9-Cbh-eGFP-KASH (Klarsicht/ANC-1/Syne-1 homology domain, hereafter AAV9-GFP)(74) to serve as a viral transduction control. This dose is comparable to doses used for P0 ICV AAV administration of Zolgensma for rescue of Δ7SMA mice, and of other base editor AAVs that enable efficient genome editing in mice(32, 74). We observed typical transduction patterns of AAV9 in the spinal cord (Fig. 3, C to E, Supplementary Text, fig. S4A)(32, 33, 75). We quantified GFP and choline acetyl-transferase (ChAT) double-positive cells in the ventral horn of spinal cords from injected mice and observed a mean transduction efficiency of 43% in spinal motor neurons (Fig. 3F), consistent with transduction efficiencies >20% previously shown to enable significant phenotypic rescue of Δ7SMA mice following ICV injection of self-complementary AAV9-SMN (Zolgensma)(32). Transduction of spinal motor neurons using 2.97x10^13^ vg/kg AAV9-GFP alone was similar (median 46%) to transduction efficiencies using the ten-fold lower concentration of 2.7x10^12^ vg/kg, suggesting that the low-dose co-transduction of AAV9-GFP accurately represents the subset of cells transduced by AAV9-ABE.

Next, we assessed base editing in transduced cells (Supplementary Text, fig. S4B). We isolated cortical nuclei of treated animals and enriched for AAV9-transduction by sorting GFP-positive cells as previously described(74, 76). We observed 87±3.5% conversion of *SMN2* C6T among transduced cells (Fig. 3G), a 2.4-fold enrichment over unsorted tissue (37%±4.7%), with high single-nucleotide precision for C6T alone (73±.2.7%) and few indels (<0.4±.1%) or bystander edits, similar to D10 editing in Δ7SMA mESCs (Fig. 2E, fig. S2H, S4C). Collectively, these data confirm that ICV injection of AAV9-ABE in Δ7SMA neonates enables efficient and precise conversion of *SMN2* C6T in the CNS of treated animals with minimal undesired byproducts(56, 57, 77).

Base editing conversion of C6T effectively converts the native *SMN2* gene to *SMN1*, thereby restoring SMN protein levels to that of wild-type cells. Current SMA drugs induce non-native SMN levels(23, 24, 32-35), and require repeated dosing or may fade over time. The permanent and precise editing of endogenous *SMN2* genes that preserves native transcript levels and native regulatory mechanisms governing *SMN* expression thus may address shortcomings of existing SMA therapies(1, 4, 5, 21, 28, 78).

### In vitro and in vivo DNA and RNA off-target analysis of ABE8e targeting SMN2 C6T

In addition to the off-target analysis in human cells described above, we also assessed the DNA and RNA specificity of the ABE strategy in mouse cells *in vitro* and *in vivo*. We performed CIRCLE-seq and validated the top 35 nominated sites in Δ7SMA mESCs (Supplementary Text, fig. S4D). We achieved 95±0.0% on-target editing at the *SMN2* transgene and only observed substantial editing at off-target site 5 in an intron of the mucin 16 gene (Muc16, 31±1.9%) that is not expressed in the CNS (fig. S4E)(79). Next, we compared this analysis to off-target editing *in vivo* following AAV9-ABE ICV injection in Δ7SMA neonates by performing verification of *in vivo* off targets (VIVO)(80). We observed between 10-27% (average 15±7%) editing at off-target site 5 in intron 54 of Muc16, and between 0.1-0.9% (average 0.5±0.3%) editing at the non-coding off-target site rank 15, compared to 87±3.5% average on-target editing of *SMN2* among GFP-positive cells in the CNS across five animals (Fig. 3G and H). These animals ranged from 4 to 18 weeks of age at the time of off-target analysis (26, 36, 42, 80, and 127 days old) and we observe no increase in off-target editing events over time. Thus, off-target editing outcomes observed in cell culture experiments were consistent with those observed *in vivo* over 18 weeks(80). The ABE strategy did not induce any detected coding mutations in either human or mouse genomes, and off-target editing *in vivo* was lower than in cell culture (~2-fold lower at Muc16 intron 54), likely due to lower copy number and expression levels in transduced cells *in vivo* or *in vivo* gene silencing over time(33, 36, 37).

Cas-independent RNA off-target adenine base editing *in vivo* is typically indistinguishable from background A-to-I conversion due to the low copy-number of ABE-expressing transgenes(33, 81). We investigated RNA off-target editing in Δ7SMA mESCs and differentiated neural lineages including motor neurons, that stably produce ABE8e from low gene copy numbers similar to those resulting from AAV9 transduction (Fig. 3I, Supplementary Text, fig. S4, F to H). Consistent with previous reports(81, 82), whole transcriptome sequencing did not reveal detected accumulation of RNA A-to-I edits over background levels of endogenous A-to-I and A-to-G changes (Fig. 3J, and fig. S4G).

Collectively, these *in vitro* and *in vivo* analyses did not reveal off-target edits of anticipated clinical or physiological significance in human or mouse cells, suggesting high target specificity of the D10 base editing approach. Continued preclinical assessment and minimization of off-target editing is important to ensure the safety of a potential base editing therapeutic for the treatment of SMA in patients.

### ABE-mediated rescue of SMA pathophysiology in mice

**Fig. 4.**
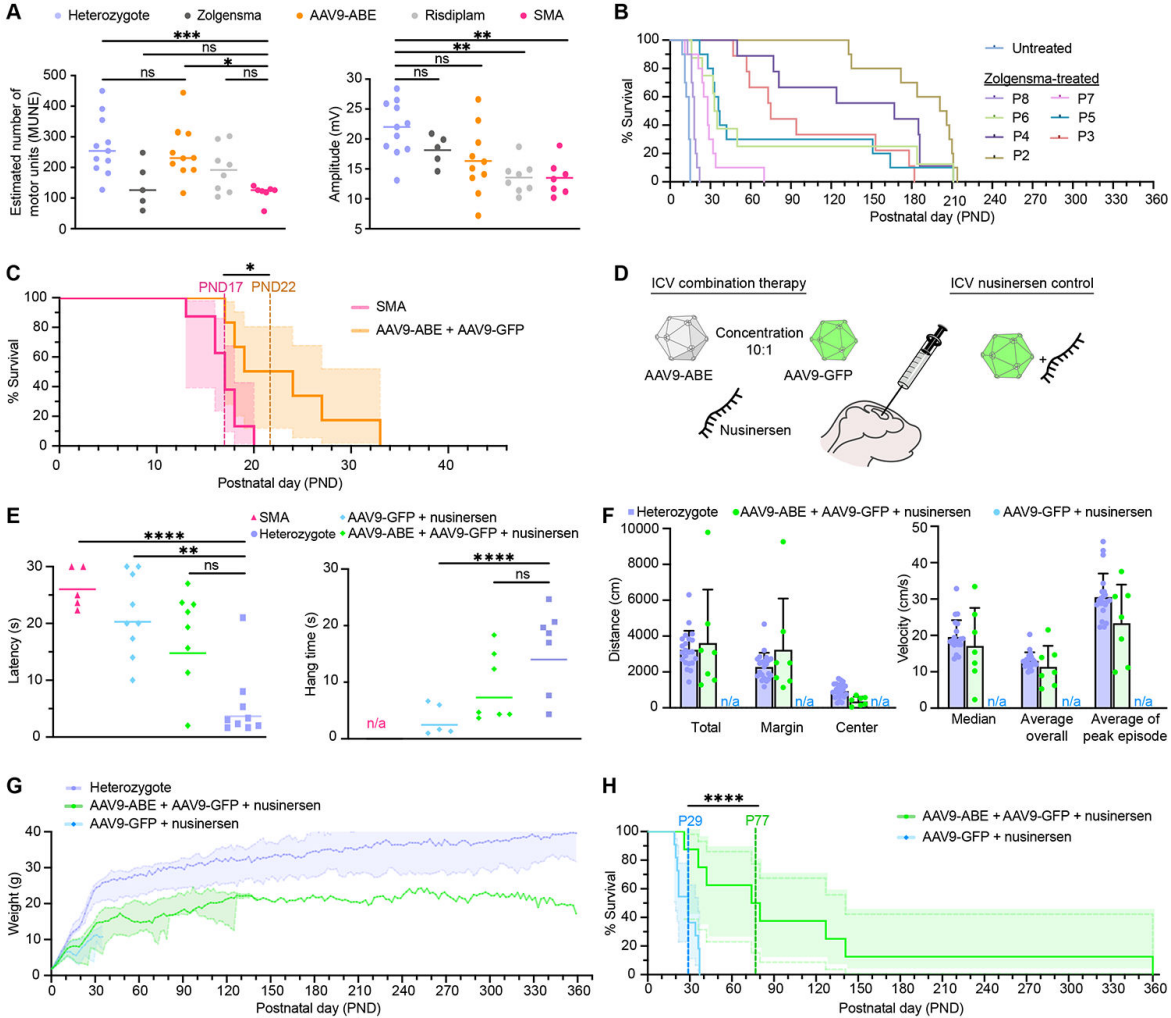
AAV9-ABE mediated rescue of Δ7SMA mice. **(A)** (Left) Motor unit number estimation (MUNE) and (Right) compound muscle action potential (CMAP) amplitude at PND12 in heterozygotes (*n*=11), and Δ7SMA mice treated with Zolgensma (*n*=5), AAV9-ABE (*n*=10), risdiplam (*n*=8), or uninjected (*n*=7). **(B)** Kaplan–Meier curve of Δ7SMA neonates ICV injected with Zolgensma from *Robbins et al. 2014* (data extracted using PlotDigitizer). Average (av), median (md), and longest (lng) survival in days: untreated (avg-13, med-14, lng-15), PND2 (avg-187, med-204, lng-214), PND3 (avg-102, med-75, lng-182), PND4 (avg-141, med-167, lng-211), PND5 (avg-76, med-37, lng-211), PND6 (avg-73, med-34, lng-211), PND7 (avg-30, med-28, lng-70), and PND8 (avg-18, med-18, lng-22).). **(C)** Kaplan-Meier curve in AAV9-ABE treated (*n*=6) and uninjected (*n*=8) Δ7SMA mice. **(D)** Neonatal ICV co-injections with AAV9-ABE, AAV9-GFP, and nusinersen. **(E)** (Left) The time required for Δ7SMA mice to right themselves in the righting reflex assay at PND7. (Right) The hang time of Δ7SMA mice in the inverted screen test at PND25. **(F)** Analysis of voluntary movement by open field tracking at PND40. (Left) Traveled distance in cm. (Right) Velocity in cm/s. **(G, H)** Body weight in grams and Kaplan-Meier curve of Δ7SMA mice. Graph line shading represents (G) standard deviation or (H) 95% CI. Animals are treated as indicated. Dots represent individual animals, *≤0.02, **≤0.01, ***≤0.005, ****≤0.001.

The physiology of AAV9-ABE treated Δ7SMA mice was improved compared to untreated animals (Movies S1 and S2). We assessed the rescue of motor phenotypes by electrophysiological measurements in AAV9-ABE treated Δ7SMA mice. We measured compound muscle action potential (CMAP) amplitude and performed motor unit number estimation (MUNE) in the gastrocnemius muscle to assess loss of motor neuron functional integrity, a key feature of SMA and preclinical SMA models(83). We compared outcomes with FDA-approved therapeutics for SMA including ICV injection of Zolgensma, and daily intraperitoneal (IP) injection of risdiplam (Evrysdi) at doses that were previously demonstrated to confer a survival benefit to these mice (2.5x10^13^ vg/kg Zolgensma and 0.1 mg/kg risdiplam, Fig. 4A)(30, 32). MUNE were reduced by 50% in untreated Δ7SMA animals compared to heterozygous mice at postnatal day (PND) 12, and Zolgensma or 0.1 mg/kg risdiplam showed little to no improvement (50% and 75% relative to heterozygotes respectively, Kruskal-Wallis test *p*>0.6). In contrast, MUNE in AAV9-ABE treated SMA mice were significantly improved compared to untreated animals (Kruskal-Wallis test p<0.02) and did not significantly differ from heterozygous animals, with values averaging 91% that of heterozygotes. CMAP amplitudes were also higher for AAV9-ABE-treated mice compared to risdiplam-treated or untreated Δ7SMA mice, while CMAP amplitudes did not significantly differ between heterozygotes, Zolgensma-treated mice, and AAV9-ABE-treated animals (Kruskal-Wallis one-way ANOVA *p*>0.2). Thus, neonatal ICV injection of AAV9-ABE measurably rescues SMA pathophysiology of spinal motor neurons.

Next, we assessed survival of ICV AAV9-ABE injected Δ7SMA mice. In SMA type I patients, therapeutic intervention can meaningfully improve disease outcomes if administered in the first several months of life(84-87), however, in Δ7SMA mice survival drops precipitously when animals receive treatment past PND6 (Fig. 4B)(88). This large difference is due in part to the highly accelerated (~150-fold greater) rate of maturation of mice compared to humans in the first month, early perinatal reduction in SMN expression that occurs in mice(89) and humans(28), and the rapid early-onset loss of motor units, which consist of spinal motor neurons and the muscle fibers that they innervate(83, 90). Restoration of SMN protein levels using inducible transgenes demonstrates that high levels of SMN are required by PND4-6 to rescue Δ7SMA mice, and delays of small numbers of days are strongly anti-correlated with survival(32, 88, 89, 91-93). In cells, complete mRNA rescue is not achieved until 7 days post D10 transfection (fig. S5A), and the time to restore SMN protein levels *in vivo* surpasses the extremely short therapeutic window in Δ7SMA mice.

The accumulation of SMN protein following transduction with the dual single-stranded AAV9 ABE8e vectors used in this study requires completion of (1) second-strand synthesis of each AAV9-ABE genome(94, 95), (2) transcription and translation of the split-intein ABE protein segments, (3) assembly and trans-splicing of the split ABE protein, (4) RNP assembly and base editing of *SMN2*, (5) transcription of full-length C6T-modified endogenous *SMN2* pre-mRNA driven by its native promoter, and (6) splicing and translation of corrected *SMN2* transcripts. Thus, the timing for SMN protein rescue following AAV9-ABE administration is slower than fast-acting splice-switching drugs or constitutive gene complementation from SMN cDNA encoded by a self-complementary AAV9-SMN vector such as Zolgensma(94-96). We recently demonstrated that *in vivo* base editing impacts protein levels by ~1-3 weeks post-administration(81).

Despite the incongruent timeline of base editing-mediated rescue for ideal rescue of Δ7SMA mice, AAV9-ABE increased the lifespan of treated animals by ~33% in two colonies in different institutions (Supplementary Text, Materials and Methods, Fig. 4C, and fig. S5, B to D). Lifespan of treated animals improved from an average of 17 days (median 17 days, maximum 20 days) to 23 days (median 22 days, maximum 33 days, Mantel-Cox test *p*<0.02,). As anticipated, the lifespan extension resulting from AAV9-ABE treatment is similar to that achieved by scAAV9-SMN gene therapy in *post-symptomatic* (>PND7) Δ7SMA mice (Fig. 4B)(32, 73, 88, 93). Collectively, these data demonstrate that postnatal conversion of *SMN2* C6T by AAV9-ABE rescues SMA motor phenotypes in mice, including the number (MUNE) and output (CMAP) of functional motor units innervating muscle, and that the prolonged process of AAV9-ABE-mediated SMN restoration results in mostly post-symptomatic rescue in Δ7SMA mice that results in a significant, but limited improvement in animal lifespan.

Upregulation of SMN protein levels improves motor function and life expectancy of SMA patients and animal models if achieved prior to onset of neuromuscular pathology and symptoms(13, 32, 86-88, 93), yet even high levels of SMN protein cannot correct neuromuscular junction defects once SMA has progressed to an advanced stage and loss of motor neurons upon cell death is irreversible. We therefore sought to extend the effective therapeutic window for gene editing by transient early administration of an existing approved SMA drug to attenuate disease progression, as has previously been applied to study milder forms of SMA in mice(73, 97, 98). Since SMA patients in a gene editing clinical trial would likely be receiving an SMA drug, repeating the base editing treatment in mice receiving an existing SMA drug would also inform a potential future clinical application of this approach.

### Combination therapy improves the lifespan of ABE-treated SMA mice

Transient SMA drug administration can ameliorate SMA pathology and extend survival of Δ7SMA mice. We hypothesized that attenuating disease progression using nusinersen could extend the unusually short therapeutic window of Δ7SMA mice and allow AAV9-ABE-mediated rescue to begin before extensive irreversible SMA damage occurs. The mechanism of nusinersen (binding to *SMN2* pre-mRNA) is orthogonal to base editing of *SMN2* genes, and co-transfection of 20 nM nusinersen did not affect base editing outcomes or inclusion of exon 7 in spliced *SMN* transcripts following D10 in Δ7SMA mESCs (fig. S5, A and E). We assessed whether co-administration of nusinersen can improve phenotypic rescue from AAV9-ABE treatment. A single ICV injection of nusinersen at PND0 has been shown to extend survival of Δ7SMA mice by several weeks(99), thus we co-injected a single low dose (1 μg) of nusinersen together with AAV9-ABE and AAV9-GFP in Δ7SMA neonates (Supplementary Text). As a control, we also treated Δ7SMA neonates with 1 μg nusinersen and AAV9-GFP but no base editor (Fig. 4D). We assessed motor coordination and overall muscle strength at PND7 using the righting reflex test, which measures the time needed for a mouse placed on its back to right itself (Fig. 4E). We observed significant difference between heterozygotes and nusinersen-treated or untreated Δ7SMA mice (Kruskal-Wallis test *p*≤0.01), but no significant difference between mice treated with combined AAV9-ABE+nusinersen compared to heterozygous littermates (Kruskal-Wallis test *p*>0.1).

Next, we assessed motor strength and coordination of treated and heterozygous mice using an inverted screen test, which measures how long the mice can hang inverted from a screen mesh surface. At PND25, Δ7SMA animals treated with nusinersen alone performed significantly worse than healthy heterozygous mice at inverted screen testing (Kruskal-Wallis test *p*=0.007, Fig. 4E). In contrast, the AAV9-ABE+nusinersen combination-treated animals showed no significant difference in the inverted screen assay from healthy heterozygous mice. Notably, half of nusinersen-only treated animals were deceased by this timepoint, and age-matched untreated Δ7SMA mice do not survive long enough for this PND25 assay.

For a more complete behavioral assessment of treated and heterozygous animals, we performed extensive multiparametric analysis of voluntary movement by open field tracking at PND40 (Fig. 4F, and fig. S5, F to J). Across 33 parameters including traveled distances, velocity, duration, and counts of various activities, the measured behaviors of AAV9-ABE+nusinersen combination-treated animals showed no significant difference with those of heterozygous mice (Mann-Whitney test *p*>0.5). Neither nusinersen-only treated or untreated age-matched Δ7SMA mice were available as reference for this PND40 assay due to their short lifespan.

We also assessed the effect of combination AAV9-ABE and nusinersen treatment on weight and lifespan of Δ7SMA mice. The weight of nusinersen-only and AAV9-ABE+nusinersen combination-treated Δ7SMA mice steadily increased and were indistinguishable for the first week of life, after which weight gain slowed in the nusinersen-only cohort (Fig. 4G). Combination-treated animals maintained on average 61±4.0% the weight of heterozygous animals throughout their lifespans. The nusinersen-only injection improved lifespan of Δ7SMA mice from an average of 17 days (median 17, maximum 20 days, Fig. 4C) to an average 28 days (median 29, maximum 37 days, Mantel-Cox test *p*=0.0001, Fig. 4H). Importantly, combination treatment of AAV9-ABE with nusinersen improved survival of Δ7SMA mice to on average of 111 days (median 77, Mantel-Cox test *p*=0.002), with over 60% of animals surviving beyond nusinersen-only controls, and a 10-fold increase in maximum lifespan (37 days maximum with nusinersen only, compared to 360 days maximum with AAV9-ABE). Combination AAV9-ABE+nusinersen-treated SMA mice also exhibited normal behavior and vitality well beyond the lifespan of nusinersen only-injected controls (P40, P96, and P200 in Movies S3 to S5). Collectively, these data indicate that transient extension of the very narrow therapeutic window in Δ7SMA mice can greatly improve phenotypic rescue of SMA from base editing of *SMN2*.

While neonatal AAV9-ABE ICV injection alone enables life extension in Δ7SMA mice that resembles >PND7 ICV injection with Zolgensma (Fig. 4B and C)(88), co-administration of 1 μg nusinersen temporarily slows disease progression and broadens the narrow therapeutic window, allowing base editing the opportunity to enable lifespan rescue that more closely resembles that of *pre-symptomatic* Zolgensma administration at ≤PND3 (Fig. 4H). Moreover, these data demonstrate compatibility of AAV9-ABE with nusinersen as a one-time treatment without evident adverse effects, and with apparent synergy to improve therapeutic outcomes. Such a combination therapy approach may play an important role in future clinical trial designs for one-time SMA treatments that permanently correct a genetic cause of the disease, and for clinical application in patients already receiving treatment.

## Discussion

Current treatment options for SMA have revolutionized care for thousands of patients, effectively extending lifespan, preventing the loss of motor function in pre-symptomatic patients, and delaying progression in symptomatic patients by increasing full-length SMN protein levels(13, 24, 86, 87, 91, 100). However, current therapies do not restore endogenous protein levels and native regulation of SMN, which could result in pathogenic SMN insufficiency in motor neurons or potential long-term toxicity in other tissues(21, 23-28, 35). Furthermore, the transient therapies nusinersen and risdiplam require repeated dosing throughout a patient’s lifetime, and it is unclear whether Zolgensma gene complementation will persist in motor neurons(36, 37). Thus, achieving permanent and endogenously regulated rescue of SMN protein levels is an important goal of a future therapeutic for SMA patients. The optimized D10 ABE strategy developed in this work is a one-time treatment that enables permanent and precise editing of endogenous *SMN2* genes while preserving native transcript levels and regulatory mechanisms that govern *SMN* expression(1, 4, 5, 28, 78, 101). As such, a future base editing therapeutic approach could offer substantial benefits over existing SMA therapies.

We compared 79 total nuclease and base editing strategies targeting five regions of *SMN2* to induce either post-transcriptional or post-translational regulatory changes in *SMN2* that upregulate SMN protein production. BE-Hive and inDelphi machine learning models enabled the design of precise editing strategies that in some cases were not obvious, and pre-selected sgRNAs for genotypic and phenotypic validation of editing outcomes. All SMA patients regardless of their *SMN1* mutations must carry the *SMN2* gene to complete gestation(7), and thus the genome editing strategies identified in this study have the potential to benefit all SMA patients.

While on-target Cas nuclease editing at *SMN2* can be precise, DSBs can result in large deletions and chromosomal rearrangements, especially when induced simultaneously at multiple genomic loci(102). Given that SMA patients usually have multiple copies of *SMN2*, nuclease editing may result in unintended restructuring of the chromosome region (5q13) that harbors *SMN* genes(103, 104). In contrast, base editors precisely convert nucleotides without inducing DSBs(50, 105, 106), and result in greater SMN protein upregulation than the nuclease strategies in this study (up to 50-fold by base editors compared to up to 17-fold by nucleases). We therefore recommend that future gene editing therapeutic strategies for SMA use base editing rather than nucleases.

ABE strategy D10 demonstrated high on-target efficiency and specificity, with minimal Cas-dependent or Cas-independent off-target DNA or RNA editing. It is possible that extended base editor expression in cells, as can result from AAV-delivery, could result in a greater accumulation of genomic and transcriptomic off-target events. Therefore, a deeper assessment of genomic and transcriptomic off-targets and efforts to minimize off-target editing risk will be important in the preclinical development of a potential base editing therapeutic for SMA. If needed, Cas-independent editing events can be further minimized by alternative delivery strategies that shorten exposure to base editors(62), and by the use of tailored deaminases such as the V106W variant of TadA*-8e(62, 64) or TadA-8.17-m(107).

SMA has variable presentation in humans that largely correlates with the copy number of *SMN2*(108-112). Type I SMA patients have two *SMN2* copies and present with symptoms within the first 6 months, type II patients have three copies and present with symptoms by 18 months, while type III patients have 3-4 *SMN2* copies with later onset. Early intervention is paramount to achieving the best outcomes for SMA patients. The window to effectively treat type II and III patients is broader than for type I patients, who ideally receive treatment within the first few months of life and up to 18 months(13, 24, 84-87, 100). Indeed, we directly observed the critical role of differences in timing on the order of days in determining the efficacy of an AAV9-ABE treatment in Δ7SMA mice, which have an unusually short (≤6 days) therapeutic window compared to the timescale of base editing (weeks)(88). We show that the FDA-approved ASO drug nusinersen can extend the very short therapeutic window for rescue in Δ7SMA mice, allowing base editing-mediated rescue of SMN protein levels to occur to a greater extent(81). We anticipate that the broader therapeutic window in human SMA patients would provide ample opportunity for AAV9-ABE-mediated restoration of SMN protein levels to take place without the need for co-administration of a transient therapeutic. Nevertheless, our study demonstrates the compatibility of base editing with nusinersen as a combination therapy approach to treat SMA in animals, which may be valuable for future clinical applications.

The ICV-injected AAV9-ABE animals in our study exhibited mouse-specific peripheral disease phenotypes that are common in SMA mouse models including necrosis of the extremities(113, 114), while exhibiting otherwise normal behavior and vitality without displays of progressive muscle weakness. However, SMA treatment that is restricted to the CNS also reveals a late onset lethal cardiac abnormality specific to Δ7SMA mice(32, 115-117), and likely underlies the sudden late-stage fatality observed in ICV AAV9-ABE treated animals in this study. Treating both CNS and peripheral tissues may ameliorate this murine cardiac phenotype to improve lifespan of treated Δ7SMA mice compared to ICV-injected animals(115, 118). Nevertheless, peripheral restoration of SMN protein does not appear to be required to rescue SMA lethality in humans in light of patients successfully treated intrathecally with Spinraza(23, 31, 84, 91, 119).

As demonstrated in this work, dual-AAV delivery of base editors supports therapeutic levels of editing in mouse models of human disease(120, 121). After these *in vivo* experiments were completed, our lab developed efficient *in vivo* base editing using single-AAV9-ABE systems that use size-minimized AAV vector components and one of a suite of small Cas protein domains that are highly active as ABEs(81). Such single-AAV base-editing systems may simplify the development of future base editor therapeutics, and potentially minimize the required dose and potential side effects of AAV in clinical settings(122).

## Materials and Methods

### Cell culture

Culture of mESCs, HEK293T, and U2OS cells was performed according to previously published protocols(123). mESCs were maintained on 0.2% gelatin-coated plates feeder-free in mESC media composed of Knockout DMEM (Life Technologies) supplemented with 15% defined fetal bovine serum (FBS, HyClone), 0.1 mM nonessential amino acids (NEAA, Life Technologies), Glutamax (GM, Life Technologies), 0.55 mM 2-mercaptoethanol (b-ME, Sigma-Aldrich), 1X ESGRO LIF (Millipore), with the addition of 2i: 5 nM GSK-3 inhibitor XV (Sigma-Aldrich), and 500 nM UO126 (Sigma-Aldrich). Δ7SMA mESCs were a kind gift from Lee L. Rubin. HEK293T cells were purchased from ATCC (CRL-3216) and were maintained in DMEM (Life Technologies) supplemented with 10% fetal bovine serum (ThermoFisher Scientific). U2OS cells were purchased from ATCC (HTB-96) and were maintained in McCoy's 5a medium (Life Technologies) supplemented with 10% fetal bovine serum (ThermoFisher Scientific). All cells were regularly tested for mycoplasma.

For genome editing experiments, cells were seeded one day prior to be ~70-80% confluent on the day of transfection and transfected with sgRNA and genome editing plasmids at a 1:1 molar ratio using Lipofectamine 3000 (ThermoFisher Scientific) in accordance with the manufacturer’s protocols. For stable integration of plasmids, cells were co-transfected with Tol2 transposase at an equimolar ratio. Cells that did not undergo antibiotic selection were cultured for 3-5 days before harvesting. For antibiotic selection, Δ7SMA mESCs were treated with 50 μg/mL hygromycin B (Life Technologies) and/or 6.67 μg/mL blasticidin as indicated, starting 24 hours after transfection. For transient selection, antibiotics were removed from the media after 48 hours. Selected cells were allowed to recover and expand prior to harvesting. All sgRNA sequences designed for this study are listed in the supplement.

For Δ7SMA mESCs nusinersen experiments, cells were transfected with 20 nM of fully 2′-O-methoxyethyl (MOE)-modified ASO (5’-TCACTTTCATAATGCTGG-3') on a phosphorothioate backbone (TriLink), using Lipofectamine 3000 (ThermoFisher Scientific). After 24 hours media was replaced every other day with fresh mESC+2i media. For splicing rescue by risdiplam, mESC media was supplemented with 0.1–1 μM of risdiplam (RG7916, Selleck Chemicals LLC) in DMSO, as indicated. Cells were harvested at the indicated timepoints.

### High-throughput sequencing of genomic DNA

Sequencing library preparation was performed according to previously published protocols(50). Primers are listed in the supplement. Briefly, we isolated genomic DNA (gDNA) with the QIAamp DNA mini kit (Qiagen) and used 250-1000 ng of gDNA for individual locus editing experiments and 20 μg of gDNA for comprehensive context library samples. Sequencing libraries were amplified in two steps, first to amplify the locus of interest and second to add full-length Illumina sequencing adapters using the NEBNext Index Primer Sets 1 and 2 (New England Biolabs) or internally ordered primers with equivalent sequences. All PCRs were performed using NEBNext Ultra II Q5 Master Mix. Samples were pooled using Tape Station (Agilent) and quantified using a KAPA Library Quantification Kit (KAPA Biosystems). The pooled samples were sequenced using Illumina NextSeq or MiSeq. Alignment of fastq files and quantification of editing frequency for individual loci was performed using CRISPResso2 in batch mode(67). The editing frequency for each site was calculated as the ratio between the number of modified reads (i.e. containing nucleotide conversions or indels) and the total number of reads. Base editing characterization library analysis was performed as previously described(50).

### Quantification of SMN splice products

We isolated mRNA from Δ7 mESCs with the RNeasy mini kit (Qiagen) and performed reverse transcription using SuperScript IV (ThermoFisher) according to the manufacturer’s protocols. For targeted *SMN2* splice product quantitation by qPCR, high-throughput sequencing, or automated electrophoresis we performed reverse transcription with random hexamers. Inclusion of *SMN2* exon 7 was quantified by automated electrophoresis using Tape Station (Agilent). For unbiased *SMN2* splice product analysis by high-throughput sequencing, we performed reverse transcription using a custom oligo-dT primer with a Read 2 Illumina sequencing stub. The pooled samples were sequenced using Illumina MiSeq. All PCRs were performed using NEBNext Ultra II Q5 Master Mix, with the addition of Sybr Green for qPCR. Primers are listed in Supplementary Table 3.

### Western Blot

Cells harvested for western blot were washed with ice-cold PBS and incubated at 4 °C for 30 min while rocking in RIPA lysis buffer (ThermoFisher) supplemented with 1 mM PMSF (ThermoFisher) and cOmplete EDTA-free protease inhibitor cocktail (Roche). Lysates were clarified by centrifugation at 12,000 rpm at 4 °C for 20 min. Lysates were normalized using BCA (Pierce BCA Protein Assay Kit) and combined with 4x Laemelli buffer (BioRad) and DTT (ThermoFisher) at a final concentration of 1 mM. We loaded 10 μg of reduced protein per gel lane and performed transfer with an iBlot 2 dry blotting system (ThermoFisher) using the following program: 20 V for 1 min, then 23 V for 4 min, then 25 V for 2 min for a total transfer time of 7 minutes. Blocking was performed at room temperature for 60 minutes with block buffer: 1% BSA in TBST (150 mM NaCl, 0.5% Tween-20, 50 mM Tris-Cl, pH 7.5). Membranes were then incubated in primary antibody diluted in block buffer for 2 hours at room temperature. After a washing, secondary antibodies diluted in TBST were added and incubated for 1 hour at room temperature. Membranes were washed again and imaged using a LI-COR Odyssey. Wash steps were 3x 5-minute washes in TBST. Primary antibodies used were mouse anti-human SMN (Proteintech 2C6D9), mouse anti-mouse and human SMN (Proteintech 3A8G1) and rabbit anti-histone H3 (Cell Signaling D1H2), secondary antibodies used were LI-COR IRDye 680RD goat anti-rabbit (#926–68071) and goat anti-mouse (#926–68070).

### Base editor characterization library assay

For characterization of the ABE8e-SpCas9 base editor, we used mouse ESCs carrying the comprehensive context library according to previously published protocols(42, 50). Briefly, 15-cm plates with >10^7^ initial cells were transfected with a total of 50 μg of p2T-ABE8e-SpCas9 and 30 μg of Tol2 plasmid to allow for stable genomic integration with Lipofectamine 3000 according to manufacturer protocols, and selected with 10 μg/mL blasticidin starting the day after transfection for 4 days before harvesting. We maintained an average coverage of ~ 300x per library cassette throughout. We collected gDNA from cells 5 days after transfection, after 4 days of antibiotic selection.

### Cloning

Base editor plasmids were constructed by replacing deaminase and Cas-protein domains of the p2T-CMV-ABE7.10-BlastR (Addgene 152989) plasmid by USER cloning (New England Biolabs)(50). Individual sgRNAs were cloned into the SpCas9-hairpin U6 sgRNA expression plasmid (Addgene 71485) using BbsI plasmid digest and Gibson assembly (New England Biolabs). Protospacer sequences and gene-specific primers used for amplification followed by HTS are listed in Supplementary Table 1. Constructs were transformed into Mach1 chemically competent E. coli (ThermoFisher) grown on LB agar plates and liquid cultures were grown in LB broth overnight at 37 °C with 100 μg/mL ampicillin. Individual colonies were validated by Templiphi rolling circle amplification (ThermoFisher) followed by Sanger sequencing. Verified plasmids were prepared by mini, midi, or maxiprep (Qiagen).

AAV vectors were cloned by Gibson assembly (NEB) using NEB Stable Competent E. coli (High Efficiency) to insert the sgRNA sequence and C-terminal base editor half of ABE8e-SpyMac into v5 Cbh-AAV-ABE-NpuC+U6-sgRNA (Addgene 137177), and the N-terminal base editor half and a second U6-sgRNA cassette into v5 Cbh-AAV-ABE-NpuN (Addgene 137178)(74).

### Neural differentiation

Differentiation of Δ7SMA mESCs was performed according to established protocols(124, 125). Briefly, Δ7SMA mESCs maintained on 0.2% gelatin-coated plates feeder-free in mESC media + 2i were plated onto irradiated mouse embryonic fibroblast (iMEF) feeders on 0.2% gelatin-coated plates in mESC media for 7 days to wean cells from 2i factors. Cells were then seeded at 10^6^ in 10-cm tissue culture treated plates for 48 hours for priming and depletion of feeders. Media was replaced with neural differentiation (ND) media composed of 1:1 DMEM:F12 and Neurobasal media (Life Technologies) supplemented with 10% knockout serum-replacement (KOSR, Life Technologies), Glutamax (GM, Life Technologies) and 0.55 mM 2-mercaptoethanol (b-ME, Sigma-Aldrich), for one hour prior to trypsinization and seeding of 2x10^6^ cells in 10-cm non-tissue culture treated dishes for 24 hours. Single cells and small early embryoid bodies (EBs) in suspension were collected and transferred to 10-cm tissue culture treated plates in fresh ND media for 24 hours. Small EBs that remained in suspension were collected and transferred to 10-cm tissue culture treated plates in fresh ND media with the addition of 1μM retinoic acid (RA, Sigma-Aldrich R2625) for caudal neural differentiation (CND), or with 1μM RA and 0.5 μM smoothened agonist (SmAg, Calbiochem 566660) for motor neuron differentiation (MND) for 72 hours. Large EBs were collected and split into two 10-cm tissue culture treated plates in neural growth (NG) media composed of 1:1 DMEM:F12 and Neurobasal media supplemented with GM, B27 (Life Technologies), and 10ng/mL human recombinant glial cell line-derived neurotrophic factor (GDNF, R&D Systems 212-GD-010) for 48 hours. EBs were monitored for *Mnx1:GFP* expression to assess motor neuron differentiation efficiency and imaged using a Zeiss inverted fluorescence microscope or collected for downstream whole transcriptome analysis.

### Whole transcriptome RNA-sequencing

Library preparation, sequencing and analysis were performed by SMART-seq2 as previously described(126). Briefly, total RNA was harvested from cells using the RNeasy Mini kit (Qiagen). First, we incubated 20 ng purified total RNA with RNase inhibitor (Clontech Takara 2313B), dNTP mix (Thermo Fisher R0192), and the 3’-RT primer (5’-AAGCAGTGGTATCAACGCAGAGTAC(T30)VN-3’) at 72 °C for 3 min to anneal the RT primer. Next, we performed first-strand synthesis using the template switching oligo (TSO): (5'-AGCAGTGGTATCAACGCAGAGTACrGrG+G-3' Exiqon, Qiagen) together with RNase inhibitor, betaine (Sigma Aldrich B0300-1VL), MgCl2 (Sigma Aldrich 1028) and Maxima RNase H-minus RT (Thermo Fisher EP0751), according to the manufacturer’s protocols. We performed pre-amplification of first-strand libraries with the ISPCR primer: 5'-AAGCAGTGGTATCAACGCAGAGT-3' using KAPA HiFi HotStart (KAPA KK2601) and SYBR green (Thermo Fisher). Whole transcriptome amplification (WTA) product was washed using DNA SPRI beads (Beckman Coulter A63881) and quantified by Agilent Tapestation. We performed Tagmentation and library preparation of 0.25 ng WTA using the Nextera XT kit (Illumina) and Nextera i7 and Nextera i5 barcoding primers. Samples were pooled and washed using washed using DNA SPRI beads and quantified by Agilent Tapestation and the KAPA Universal Library Quantification kit (Roche KK4824). Libraries were run on Illumina NextSeq 550.

FASTQs were generated using bcl2fastq v2.20. Trim Galore v0.6.7 in paired-end mode with default parameters to remove low-quality bases, adapter sequences, and unpaired sequences. Trimmed reads were aligned to the GENCODE mouse reference genome M31 (GRCm39) using STAR (v2.7.10a), quantified using kallisto(127), and refined to canonical coding sequences using CCDS release 21(128). For RNA A-to-I off-target analysis, REDItools v1.3 was used to quantify the average frequency of A-to-I editing among all sequenced adenosines in each sample(129), excluding adenosines with read depth <10 or read quality score <30. The transcriptome-wide A-to-I editing frequency was calculated independently for each biological replicate as: (number of reads in which an adenosine was called as a guanosine)/(total number of reads covering all analyzed adenosines).

### Purification of SpyMac Cas nuclease protein

SpyMac Cas nuclease protein was cloned into the expression plasmid pD881-SR (Atum, Cat. No. FPB-27E-269). The resulting plasmid was transformed into BL21 Star DE3 competent cells (ThermoFisher, Cat. No. C601003). Colonies were picked for overnight growth in terrific broth (TB)+25 μg/mL kanamycin at 37 °C. The next day, 2 L of pre-warmed TB were inoculated with overnight culture at a starting OD_600_ of 0.05. Cells were shaken at 37 °C for about 2.5 hours until the OD_600_ was ~1.5. Cultures were cold shocked in an ice-water slurry for 1 hour, following which L-rhamnose was added to a final concentration of 0.8% to induce. Cultures were then incubated at 18 °C with shaking for 24 hours to produce protein. Following induction, cells were pelleted and flash-frozen in liquid nitrogen and stored at −80 degrees. The next day, cells were resuspended in 30 mL cold lysis buffer (1 M NaCl, 100 mM Tris-HCl pH 7.0, 5 mM TCEP, 20% glycerol, with 5 tablets of cOmplete, EDTA-free protease inhibitor cocktail tablets (Millipore Sigma, Cat. No. 4693132001). Cells were passed three times through a homogenizer (Avestin Emulsiflex-C3) at ~18,000 psi to lyse. Cell debris was pelleted for 20 minutes using a 20,000 g centrifugation at 4 °C. Supernatant was collected and spiked with 40 mM imidazole, followed by a 1-hour incubation at 4 °C with 1 mL of Ni-NTA resin slurry (G Bioscience Cat. No. 786-940, prewashed once with lysis buffer). Protein-bound resin was washed twice with 12 mL of lysis buffer in a gravity column at 4 °C. Protein was eluted in 3 mL of elution buffer (300 mM imidazole, 500 mM NaCl, 100 mM Tris-HCl pH 7.0, 5 mM TCEP, 10% glycerol). Eluted protein was diluted in 40 mL of low-salt buffer (100 mM Tris-HCl, pH 7.0, 1 mM TCEP, 20% glycerol) just before loading into a 50 mL Akta Superloop for ion exchange purification on the Akta Pure25 FPLC. Ion exchange chromatography was conducted on a 5 mL GE Healthcare HiTrap SP HP pre-packed column (Cat. No. 17115201). After washing the column with low-salt buffer, the diluted protein was flowed through the column to bind. The column was then washed in 15 mL of low salt buffer before being subjected to an increasing gradient to a maximum of 80% high salt buffer (1 M NaCl, 100 mM Tris-HCl, pH 7.0, 5 mM TCEP, 20% glycerol) over the course of 50 mL, at a flow rate of 5 mL per minute. 1-mL fractions were collected during this ramp to high-salt buffer. Peaks were assessed by SDS-PAGE to identify fractions containing the desired protein, which were concentrated first using an Amicon Ultra 15-mL centrifugal filter (100-kDa cutoff, Cat. No. UFC910024), followed by a 0.5-mL 100-kDa cutoff Pierce concentrator (Cat. No. 88503). Concentrated protein was quantified using a BCA assay and determined to be 12.6 milligrams per milliliter (ThermoFisher, Cat. No. 23227).

### CIRCLE-seq off-target editing analysis

Off-target analysis using CIRCLE-seq was performed as previously described(68, 130). Briefly, genomic DNA from HEK293T cells or NIH3T3 cells was isolated using Gentra Puregene Kit (Qiagen) according to manufacturer’s instructions. Purified genomic DNA was sheared with a Covaris S2 instrument to an average length of 300 bp. The fragmented DNA was end repaired, poly-A tailed, and ligated to an uracil-containing stem-loop adaptor using the KAPA HTP Library Preparation Kit, PCR Free (KAPA Biosystems). Adaptor ligated DNA was treated with Lambda Exonuclease (NEB) and *E. coli* Exonuclease I (NEB), then with USER enzyme (NEB) and T4 polynucleotide kinase (NEB). Intramolecular circularization of the DNA was performed with T4 DNA ligase (NEB) and residual linear DNA was degraded by Plasmid-Safe ATP-dependent DNase (Lucigen). *In vitro* cleavage reactions were performed with 250 ng of Plasmid-Safe ATP-dependent DNase-treated circularized DNA, 90 nM of SpyMac Cas9 nuclease protein, Cas9 nuclease buffer (NEB) and 90 nM of synthetic chemically modified sgRNA (Synthego), in 100 μl. Cleaved products were poly-A tailed, ligated with a hairpin adaptor (NEB), treated with USER enzyme (NEB), and amplified by PCR with barcoded universal primers NEBNext Multiplex Oligos for Illumina (NEB), using Kapa HiFi Polymerase (KAPA Biosystems). Libraries were sequenced with 150-bp paired-end reads on an Illumina MiSeq instrument. CIRCLE-seq data analyses were performed using open-source CIRCLE-seq analysis software and default recommended parameters (https://github.com/tsailabSJ/circleseq).

### Husbandry of Δ7SMA mice

All experiments in animals were approved by the Institutional and Animal Care and Use Committee of the Broad Institute of MIT and Harvard and Ohio State University (OSU). Δ7SMA heterozygous mice (*Smn*^+/−^; *SMN2*^+/+^; *SMNΔ7*^+/+^) were purchased from the Jackson Laboratory (005025)(55), and maintained in the Broad Institute and OSU vivaria according to recommendations in the Guide for the Care and Use of Laboratory Animals of the National Institutes of Health. Pairs of Δ7SMA heterozygotes were crossed to generate Δ7 SMA mice (*Smn*^−/−^; *SMN2*^+/+^; *SMNΔ7*^+/+^). On date of birth (PND0), pups were microtattooed on the foot pads (Aramis) with animal-grade permanent ink (Ketchum) using a sterile hypodermic needle (BD) to enable identification of individual pups. Subsequently, biopsies of ~1 mm tissue were taken from the tail using a sterile blade, lysed for genomic DNA extraction, and used for genotyping by PCR. Litter size was controlled to five pups, including 1-3 homozygous mutants, by culling and cross-fostering among same-age mice. Mice of both sexes were included in the study, although sex has been reported to not have a substantial impact on the phenotype of SMA mice (Treat-NMD SOP Code: SMA_M.2.2.003).

Electrophysiology experiments were performed at OSU. All other animal studies were performed at the Broad Institute unless indicated otherwise in the text. At the Broad Institute, the mean birthweight of heterozygous animals was 1.7±0.1 grams, and 1.5±0.1 grams for SMA pups, and any animal weighing <1.5 grams at time of birth was excluded from the study. The average weight of SMA neonates at injection on PND0 at the Broad Institute was 1.6±0.2 grams. At OSU, the mean birthweight of heterozygous animals on the day of birth was 1.3±0.1 grams and 1.2±0.1 grams for SMA pups, and any SMA, heterozygous or wild-type pup weighing ≤1.0 grams at time of birth were excluded from the study. The average weight of SMA neonates at injection on PND0 at OSU was 1.3±0.13 grams. By facility, each litter was subjected to the same exclusion criterion (Treat-NMD SOP Code: SMA_M.2.2.003). Cohort sizes were chosen based on prior experience with these animals, known to allow for determination of statistical significance. Animals were monitored daily for morbidity and mortality and weighed every other day from day of birth.

### Intracerebroventricular injections

Neonatal ICV injections were performed as previously described(74, 131). Briefly, glass capillaries (Drummond 5–000-1001-X10) were pulled to a tip diameter of approximately 100 μm. High-titer qualified AAV was obtained through the Viral Vector Core at UMass Medical School and concentrated using Amicon Ultra-15 centrifugal filter units (Millipore), quantified by qPCR (AAVpro Titration Kit v.2, Clontech), and stored at 4 °C until use. For injection, a small amount of Fast Green was added to the AAV injection solution to assess ventricle targeting. The injection solution was loaded via front-filling using the included Drummond plungers. Δ7SMA pups were anesthetized by placement on ice for 2–3 minutes, until they were immobile and unresponsive to a toe pinch. Up to 4.5 μL of injection mix was injected freehand into each ventricle on PND0-2.

### Immunofluorescence imaging of spinal cord sections

For immunofluorescence staining of transduced spinal motor neurons, Δ7SMA mice were perfused at 25 weeks with ice-cold PBS and ice-cold 4% PFA, the CNS was exposed, and the whole carcass was fixed overnight in 4% PFA. Whole spinal cord was isolated and fixed in 4% PFA overnight, then consecutively transferred to 10%, 20%, and 30% sucrose in three overnight incubations before embedding in OCT for long-term storage at –80°C. Embedded tissue was cryo-sectioned and stained with goat anti-Choline Acetyltransferase (Millipore AB144P), mouse anti-NeuN (EMD Millipore MAB377), mouse anti-GFAP (Sigma-Aldrich MAB3402), rabbit anti-GFP (Thermo scientific A-11122), and Alexa-Fluor secondary antibodies (Life Technologies), and imaged on an SP8 confocal microscope (Leica).

### Nuclear isolation and sorting of tissues

Tissue harvest and nuclear isolation was performed as previously described(74). Briefly, deceased Δ7SMA mice were stored at −80 °C until dissection of the brain and spinal cord tissue. For isolation of the cortex and cerebella were separated from the brain postmortem using surgical scissors. Hemispheres were separated using a scalpel and the cortex was separated from underlying midbrain tissue with a curved spatula. For nuclear isolation, dissected tissue was homogenized using a glass dounce homogenizer (Sigma D8938) (20 strokes with pestle A followed by 20 strokes with pestle B) in 2 mL ice-cold EZ-PREP buffer (Sigma NUC-101). Samples were incubated for 5 minutes with an additional 2 mL EZ-PREP buffer. Nuclei were centrifuged at 500 g for 5 minutes, and the supernatant removed. For spinal cord tissue, wash steps were repeated ten times. Samples were resuspended with gentle pipetting in 4 mL ice-cold Nuclei Suspension Buffer (NSB) consisting of 100 μg/mL BSA and 3.33 μM Vybrant DyeCycle Ruby (ThermoFisher) in PBS and centrifuged at 500 g for 5 minutes. The supernatant was removed and nuclei were resuspended in 1–2 mL NSB, passed through a 35 μm strainer, and sorted into 200 μL Agencourt DNAdvance lysis buffer using a MoFlo Astrios (Beckman Coulter) at the Broad Institute flow cytometry core. All steps were performed on ice or at 4 °C. Genomic DNA was purified according to the DNAdvance (Agencourt) instructions for 200 μL volume.

### Behavioral assays

Righting reflex was recorded on PND7 by placing neonates on their backs and recording the duration to right themselves with a stopwatch up to a maximum of 30 sec. For inverted screen testing, juvenile mice were subjected to the horizontal grid test for mice (Maze Engineers) on PND25 by placing the animals on a wire-mesh screen which the mice are capable of gripping, then inverting the screen over the course of 2 seconds, animal head first, over a padded surface made of bedding 4-5 cm high. The time for the animal to fall onto the bedding was recorded with a stopwatch. Each mouse was assessed with three measurements. The procedure is concluded when the animal falls onto the bedding, or if the animal exceeds 120 seconds for the measurement, in which case the screen is reverted so that the mouse is upright, and the mouse is manually removed from the screen.

Voluntary movement of adult mice is recorded on PND40 by open field testing (Omnitech Electronics). Mice were brought into the testing room under normal lighting conditions and allowed 30-60 minutes of acclimation. The animals were placed into the locomotor activity chamber with infrared beams crossing the X, Y and Z axes that plot their ambulatory and fine motor movements and rearing behavior. Recordings are analyzed using Fusion 5.1 SuperFlex software.

### Electrophysiological Measurements

Compound muscle action potential (CMAP) and motor unit number estimate (MUNE) measurements were performed as previously described(132). Briefly, at PND12 the right sciatic nerve was stimulated with a pair of insulated 28-gauge monopolar needles (Teca, Oxford Instruments Medical, NY) placed in proximity to the sciatic nerve in the proximal hind limb. Recording electrodes consisted of a pair of fine ring wire electrodes (Alpine Biomed, Skovlunde, Denmark). The active recording electrode (E1) was placed distal to the knee joint over the proximal portion of the triceps surae muscle and the reference electrode (E2) over the metatarsal region of the foot. A disposable strip electrode (Carefusion, Middleton, WI) was placed on the tail to serve as the ground electrode. For CMAP, supramaximal responses were generated maintaining stimulus currents <10 mA and baseline-to-peak amplitude measurements made.

For MUNE, an incremental stimulus technique similar to a previously described procedure was used(132). Submaximal stimulation was used to obtain ten incremental responses to calculate the average single motor unit potential (SMUP) amplitude. The first increment was obtained by delivering square wave stimulations at 1 Hz at an intensity between 0.21 mA to 0.70 mA to obtain the minimal all-or-none SMUP response. If the initial response did not occur with stimulus intensity between 0.21 mA and 0.70 mA, the stimulating cathode position was adjusted either closer or farther away from the position of the sciatic nerve in the proximal thigh to decrease or increase the required stimulus intensity, respectively. This first incremental response was accepted if three duplicate responses were observed. To obtain the subsequent incremental responses the stimulation intensity was adjusted in 0.03 mA steps and incremental responses were distinguished visually in real-time to obtain nine additional increments. To be accepted, each increment was required to be: (1) observed for a total of three duplicate responses, (2) visually distinct from the prior increment, and (3) at least 25 μV larger than the prior increment. The peak-to-peak amplitude of each individual incremental response was calculated by subtracting the amplitude of the prior response. The ten incremental values were averaged to estimate average peak-to-peak single motor unit potential (SMUP) amplitude. The maximum CMAP amplitude (peak-to-peak) was divided by the average SMUP amplitude to yield the MUNE.

### Statistical Analysis

Welch’s two-tailed t-tests were used to compare sequencing, splicing, mRNA levels, and immunostaining data. Error bars represent standard deviations of ≥3 independent biological replicates. Root mean squared error (RMSE) and Pearson’s *r*-correlation were used for correlation analysis of predicted and observed genome editing outcomes, where appropriate. Kruskal-Wallis tests were used to compare physiology measurements and behaviors of mouse cohorts under experimental conditions. Mann-Whitney tests were used to compare multiparametric measurements of voluntary behaviors of mouse cohorts. The logrank Mantel-Cox test Kaplan-Meier survival curves. All statistical tests were calculated by GraphPad Prism 9.4.1 and Microsoft Excel v16.64.

## Supplementary Material

Fig S1

Fig S2

Fig S3

Fig S4

Fig S5

Video 1

Video 2

Supplementary Material

Video 3

Video 4

Video 5
