# Supplementary material for: Base editing rescue of spinal muscular atrophy in cells and in mice
Source: Science. Author manuscript; Available in PMC 2023 Jun 15. (PMC10270003; doi:10.1126/science.adg6518)
Supplement: Supplementary Material [file NIHMS1898602-supplement-Supplementary_Material.docx]

**Supplementary Text**

*Editing SMN2 exon 8 increases full-length SMN and SMNΔ7mod protein production*

C-terminal extension SMNΔ7 proteins with five or more heterologous amino acids improves protein stability, and significantly rescue survival and motor phenotypes of severe SMA mice(*48*, *133*). We hypothesized that Cas nuclease-mediated disruption within the first five codons of exon 8, which include the EMLA and stop codons, could yield similar stabilized modified SMNΔ7 (SMNΔ7mod) protein with therapeutic potential (strategy B, **Fig. 1E**).

We used inDelphi to predict nuclease editing outcomes at *SMN2* exon 8 (**Fig. 1E**) and identified nine spacer sequences that enable the translation of five or more alternative terminal amino acids from exon 8 (‘predicted % precision’). After considering the PAM compatibility of these spacers with SpCas9-variant nucleases (‘predicted % PAM efficiency’, strategies B1-B16)(*43*, *44*, *52*), we selected eight for further validation (B1, B5-7, B9-11, B16). Five of these strategies edited *SMN2* exon 8 with average 98±3.6% efficiency and induced 48–76% desired alleles (B6, B7, B9, B10, B11, **Fig. 1E**) that caused a significant increase in SMN protein stability of 5.6-fold on average relative to untreated cells, up to a maximum of 7.0-fold by B11 (Welch’s two-tailed t-test *p*=0.007, **Fig. 1F, and fig. S1B**). Collectively, these data demonstrate that predictive modeling of indel products can facilitate the design of non-obvious gain-of-function alleles in *SMN2* to improve mRNA splicing and protein stability (**fig. S1C**).

Nuclease-mediated editing of exon 8 near the 3’ SA by B1 improved SMN protein stability more than anticipated (9.1-fold) based on its induction of SMNΔ7mod proteins (35±14% desired alleles, **Fig. 1, E and F**). We tested whether disrupting splicing of exon 8 using a nuclease or cytosine base editor directly improved SMN protein stability (C-nuc and C-CBE), and found that both strategies significantly increased SMN levels (3.3-fold and 9.5-fold, respectively **Fig. 1H, and fig. S1D**). To investigate how exon 8 SA disruption affects *SMN2* transcripts, we performed reverse transcription quantitative PCR (RT-qPCR) to measure the abundance of *SMN2* mRNA after editing. The terminal sequence of *SMN2,* including intron 7 and the 3’ untranslated region (UTR) of exon 8, encode negative regulators of gene expression and mRNA transcript stability(*18*, *134*). Alternative splicing at the terminus of *SMN2* could therefore increase SMN levels. We amplified a region from exon 4 through 6 that should be unaffected by alternative splicing at the terminus of *SMN2* transcripts and observed a 1.5-fold increase in *SMN2* transcripts following C-CBE editing compared to an unrelated sgRNA control (**fig. S1H**). Still, the substantial (9.5-fold) increase in SMN levels following C-CBE editing suggests that additional mechanisms may contribute to the increased abundance of SMN protein.

We speculated that alternative splice isoforms of *SMN2* yield more stable SMN protein products following exon 8 SA editing. To test this possibility, we performed high-throughput sequencing on amplified *SMN2* transcripts from treated and control Δ7SMA mESCs, and observed a profound shift in *SMN2* splice products (**Fig. 1I**). Splicing at the 5’ (5-prime)-end of exon 8 was reduced 2.1-fold by C-CBE (40±2.8%) and 1.1-fold by C-nuc (78±1.2%) compared to untreated cells (85±0.5%). Splicing to cryptic splice sites was rare in products that do not contain exon 7, and these transcripts encode the alternative translation of 16 and 33 C-terminal amino acids (≤0.27% splicing to exon 6B and ≤2.6% splicing to alternative downstream acceptors, respectively(*135*–*137*)) which are likely to result in stable SMNΔ7mod protein isoforms(*48*, *133*, *135*–*137*). More importantly, editing increased inclusion of exon 7 by 2-fold with C-CBE (63±2.0%) and 1.6-fold with C-nuc (50±1.1%) relative to untreated cells (24±1.4%). Transcripts that include exon 7 either splice near the disrupted splice acceptor of exon 8, or to cryptic exons 9 and 9tr1(*138*), or they retain intron 7 as occurs in some functional transcript variants of *SMN2* (ENST00000511812.5). Importantly, all transcripts that include exon 7 encode full-length SMN protein and can therefore complement loss of *SMN1*, and intron 7-retaining transcripts were greatly increased by C-CBE and C-nuc editing (31-fold and 12-fold, respectively) compared to untreated cells (Welch’s two-tailed t-test *p* ≤0.02, **fig. S1I**). Thus, the substantial increase in SMN protein levels following exon 8 SA editing predominantly arises from an increase in full-length SMN, with a smaller contribution from SMNΔ7mod products with increased stability.

Collectively, the *SMN2* editing strategies tested here permanently increase SMN protein levels up to 17-fold (strategy A2), 9.1-fold (strategy B1), and 9.5-fold (strategy C-CBE). As a 1.5- to 2-fold increase in SMN protein is therapeutic for SMA patients(*23*, *24*), these strategies represent promising approaches for further therapeutic studies.

inDelphi and BE-Hive predictive modeling of SpCas9-variant nucleases and base editors

Computational models can enable accurate predictions of Cas nuclease indel frequencies and facilitate the design of efficient and precise genome editing experiments(*42*, *139*–*142*). The data used to develop such models is predominantly generated using wild-type Cas nucleases. In this study we performed a comprehensive comparison of inDelphi predictions with empirical editing and phenotypic outcomes (**Fig 1 B to I**). By computational identification of spacer sequences that can enable the formation of desired indel products, we narrow down the number of strategies for downstream splicing and protein analysis.

The existing variety of PAM-variant Cas9 proteins is greatly enabling for site-specific genome editing(*43*, *44*, *52*, *143*, *144*), however, there are no inDelphi-equivalent models for all of these proteins. We investigated whether inDelphi predictive models of wild-type SpCas9 editing outcomes accurately reflect the observed indel frequencies induced by SpCas9 PAM variants to assess the generalizability of inDelphi for Cas variants. We compared the frequencies of edited genotypes that inDelphi predicts for a given sgRNA against the observed edited products induced by SpCas PAM-variant nucleases at the *SMN2* ISS-N1 and exon 8 loci in Δ7 SMA mESCs. We observed comparable predictive power for wild-type SpCas9 and iSpyMac (**Fig. 1, B and E, fig. S1C**, root mean squared error, RMSE*=1.24* and *1.62*) and moderate predictive ability for engineered SpCas9 PAM-variant family nuclease editors (Cas9-NG, SpG, SpRY, RMSE*=2.67*). These data support that inDelphi computational predictions can aid in the design of genome editing experiments using SpCas9 PAM-variant nucleases. Similarly, we assessed the accuracy of BE-Hive models trained on SpCas9 base editors to predict editing outcomes of SpCas9-variant adenine and cytosine base editors (C-CBE, D1-D19, E1-E23, **Fig. 1G, 2C, and fig. S2G**), and found that they do so with high accuracy (**Fig. 2D**).

The inDelphi and BE-Hive models can predict the relative frequency of edited genotypes from SpCas9-variant editors. The absolute frequency of edited genotypes is determined by the editing efficiency of a given strategy that is dependent on the affinity of the Cas ribonucleoprotein (RNP) complex for a given locus. A key driver of Cas RNP efficiency is the PAM-compatibility of the Cas protein for the target locus. In this study, we made *a priori* estimations of editing efficiencies for 36 nuclease and 39 base editing strategies that use SpCas9-variants based on their PAM affinity that is reported in the literature(**Fig. 1, B and E, 2C, and fig. S2G**)(*43*–*45*, *52*, *143*). We observed nuclease and base editing efficiencies that were only partially consistent with these estimates. Notably, certain sgRNA+nuclease combinations edited with substantially lower efficiency than anticipated based on reported PAM compatibilities. For example, while sgRNAs for strategies A5-6 and for A13-14 each have an ‘NAAT’ PAM, both SpRY and iSpyMac performed significantly worse with the A13-14 sgRNA (average 84% for A5-6 and 18% for A13-14, Welch’s two-tailed t-test *p<0.02*), confirming that additional factors play a strong role in determining the on-target activity of these nucleases. Thus, a deeper understanding of sgRNA, PAM, and Cas protein determinants of nuclease activity is needed to accurately predict the editing efficiency of evolved and engineered Cas9 variants.

*Predictive modeling of ABE8e base editing outcomes*

*SMN2* splicing is strongly affected by single-nucleotide changes in exon 7(*51*). We asked whether precise base editing of single nucleotides in *SMN2* exon 7 splicing regulatory elements (SREs), such as the master regulator of splicing at position 6 of intron 7 (C6T), could elevate full-length SMN production (strategy D, **Figs. 1A** and **2A**) even more potently and with less genotypic outcome heterogeneity than strategies A, B, and C. BE-Hive machine learning models trained on large-scale ABE7.10 base editing data predict that *SMN2* C6T editing was largely intractable to base editing with ABE7.10 (**fig. S2, A and B**)(*50*), as confirmed by a recent report of 3-5% optimized editing of this site using ABE7.10(*145*). The inefficient editing of this target can be attributed to a lack of canonical NGG-PAM sequences that position C6T within the editing window of SpCas9-ABE7.10(*50*, *105*) (**Fig. 2B**), and the reduced compatibility of the ABE7.10 deaminase with Cas protein PAM-variants(*54*, *62*).

Using phage-assisted continuous evolution (PACE)(*62*), we recently developed ABE8e, which uses a deoxyadenosine deaminase evolved from ABE7.10 for increased activity and greater compatibility with a wide range of Cas protein variants(*146*). We sought to deepen our understanding of the factors that govern ABE8e editing outcomes using our previously reported BE-Hive ‘comprehensive context library’ in mESCs(*50*)–a stably integrated library of 10,638 matched sgRNA and target pairs that includes 8,142 target sequences with all possible 6-mers surrounding a substrate A or C nucleotide at protospacer position 6, and 2,496 sequences that collectively contain all possible 5-mers across positions -1 to 13 (**fig. S2C**). We observed a high correlation of ABE8e base editing outcomes across two biological replicates (Pearson *r=*0.86), and greater average base editing activity (frequency of target-modified outcomes among total sequenced reads) compared to ABE7.10 and ABE7.10-CP1041 across all sites (66% for ABE8e, compared to 12% for ABE7.10 and 20% for ABE7.10-CP1041 in a prior analysis(*50*)). We also observed significantly altered sequence-activity characteristics relative to ABE7.10, including a broadened editing window spanning protospacer positions 3-10 (here defined as ≥30% of maximum editing, **fig. S2, D and E**)(*50*, *62*, *105*). The evolved ABE8e deaminase maintains a dislike for bystander adenines surrounding a target A, although this effect is outweighed by a general increase in enzymatic activity(*146*). Finally, ABE8e has an exceptionally high ratio of base edits relative to indels of 817:1 (BE:indel ratio, geometric mean).

These characterizations refine and expand upon previous characterizations of ABE8e base editing activity(*62*). We used the resulting data to train BE-Hive machine learning models to predict bystander editing patterns and base editing efficiency of ABE8e(*50*), freely accessible as part of the BE-Hive suite at www.crisprbehive.design.

*Off-target analysis of the ABE strategy in the human and mouse genome*

To further investigate Cas-dependent off-target editing of the ABE strategy in the human genome with higher sensitivity than with ABE8e, we assessed off-target editing at the top 23 CIRCLE-seq-nominated loci in HEK293T cells using SpyMac nuclease and the *P8* sgRNA (**fig. S3I**). We observed indels at 42±3.0% of *SMN2* alleles, 2.1±0.3% of *SMN1* alleles and up to 0.22% editing above background at nine off-target loci (averaging 0.08±0.07%), with no detected indels at the remaining sites. Thus, the SpyMac Cas protein paired with the *P8* sgRNA used in the ABE strategy is highly specific to the *SMN2* on-target locus, and thereby greatly contributes to the high genomic specificity of the ABE strategy.

We also assessed the DNA specificity of the ABE strategy in mouse cells. We performed CIRCLE-seq using mouse genomic DNA extracted from NIH3T3 cells *in vitro* with SpyMac nuclease+*P8* sgRNA RNPs. We identified 108 candidate DNA off-targets at primarily intergenic and intronic loci, in addition to four coding loci (off-target rank 32, 33, 37 and 86, **fig. S4D**). We then validated nominated DNA off-targets in cell culture by measuring ABE-mediated editing at the top 35 CIRCLE-seq nominated hits in Δ7SMA mESCs. We observed 95±0.0% on-target editing at the *SMN2* transgene (**fig. S4E**), and among the 35 nominated sites assayed we detected substantial off-target editing only at off-target site 5, located within intron 54 of the mucin 16 gene (Muc16, 31±1.9%) which is not expressed in the CNS(*79*, *147*), and minimal editing (between 0.1-0.5%) at five additional non-coding loci. *In* vivo, we observed lower off-target editing than in cell culture (~2-fold lower at Muc16 intron 54, (**Fig. 3G and H**), likely due to lower copy number and expression levels in transduced cells *in vivo* or *in vivo* gene silencing over time(*33*, *36*, *37*), compared to brief *in vitro* cell culture transfection experiments.

*Off-target analysis of the ABE strategy in the mouse transcriptome*

Adenine base editing can induce RNA off-target deamination in a Cas-independent manner(*62*, *64*–*66*, *148*, *149*). These events are rare in *in vitro* transient transfection conditions where the copy number of base editors is in the hundreds(*62*, *64*, *105*, *107*, *149*). *In vivo*, these events are often indistinguishable from endogenous background A-to-I deamination rates by either whole transcriptome analysis or by deep sequencing of individual abundant RNA transcripts due to the to low single-digit copy number of ABE8e transgenes mediated by AAV delivery(*33*, *81*, *150*). However, endogenous A-to-I frequencies and transcriptomes differ by cell type and may thus affect the relative burden of ABE8e in some cells.

To investigate RNA off-target editing across more cell types that stably express ABE8e, we performed stable integration of our ABE strategy in Δ7SMA mESCs using Tol2 which induces ~1 to 5 integrations of the full-length ABE8e editor per cell(*151*), similar to *in vivo* transgene copy numbers following dual-AAV9 delivery of our split-intein ABE8e base editor deaminase (~1 to 6 copies)(*33*). Next, we differentiated untreated and stable D10 expressing Δ7SMA mESCs towards motor neurons and caudal-neural lineages by activation of caudalizing retinoic acid (RA) and ventralizing sonic hedgehog (*Shh*) pathways in embryoid bodies (EBs) according to established protocols (**Fig. 3I**)(*124*). Fluorescence microscopy revealed strong *Mnx1*:*GFP* expression and axon elongation in the bulk of the motor neuron differentiated (MND) population, and moderate reporter expression and axon extension in a minority of caudal-neural differentiated cells (CND), as anticipated (**fig. S4F**)(*124*).

We isolated RNA from Δ7SMA mESCs and differentiated MND and CND populations for reverse transcription and measured the abundance of ABE8e by RT-qPCR that confirm stable expression of the ABE strategy is maintained (**fig. S4G**). Next, we performed whole transcriptome analysis by RNA-seq. Gene expression analysis revealed the expression of various motor neuron specific, neuron specific, spinal cord patterning, glia, and embryonic stem cell markers in ESC, MND, and CND populations (**fig. S4H**), in agreement with prior characterizations(*124*, *152*). We assessed off-target ABE8e editing events across the transcriptome and did not observe significant accumulation RNA A-to-I edits in ABE8e expressing populations over background levels of A-to-I and A-to-G changes (**Fig. 3J**), similar to previous studies(*81*, *82*, *150*).

These data demonstrate that our ABE strategy does not significantly contribute to the overall A-to-I burden across the transcriptome of targeted cells. Deep assessment of individual transcripts may uncover specific RNA A-to-I changes induced by adenine base editors that could impact cell function, and therefore continued assessment and minimization of genomic and transcriptomic off-targets is critical in the pre-clinical development of future base editing therapeutics(*62*, *64*, *107*).

*In vivo targeting of AAV9-ABE in Δ7SMA mice*

Although base editor and sgRNA expression cassettes typically exceed the packaging capacity of a single AAV vector, we and others have previously demonstrated that co-delivery of split-base editors packaged into two AAVs can overcome this packaging limitation(*74*, *120*, *153*). Fusing trans-splicing inteins to each half of a base editor split within the Cas9 domain enables efficient self-assembly of a full-length base editor when each half is co-expressed in cells. We designed dual-AAV ABE vectors using split DnaE intein halves from Nostoc punctiforme (Npu), dividing ABE8e-SpyMac within the SpCas9 domain immediately before Cys 574 (**Fig. 3A**), similar to the architecture of our previously reported v5 AAV-ABEmax(*74*, *154*).

We included one *P8* sgRNA expression cassette on the C-terminal encoding AAV vector, as in the architecture of v5 AAV-ABEmax(*74*). Since ABE8e base editors only encode one evolved TadA* monomer, they are smaller (4.8kb) than ABEmax (5.4kb) base editors, which encode a wtTadA-TadA* heterodimer(*62*, *105*). This 600-bp reduction in the N-terminal encoding AAV vector enables inclusion of a second sgRNA expression cassette on the N-terminal encoding AAV (v6 AAV-ABE8e, **Fig. 3A**). In Δ7SMA mESCs, co-transfection of the v6 dual-AAV plasmids encoding the split-intein ABE strategy results in *SMN2* C6T base editing with similar efficiency to full-length ABE8e-SpyMac transfection (**fig. S3J**). We refer to the v6 dual-AAV split-intein ABE strategy as ‘AAV-ABE’ hereafter.

Neonatal ICV injections of 2.7x10^13^ vg/kg of the dual AAV9-ABE vectors with 2.7x10^12^ vg/kg of AAV9-GFP resulted in typical robust transduction of non-dividing cells in the CNS. In the spinal cord, GFP signal is observed in the ventral and dorsal horns, overlapping with NeuN+ staining of post-mitotic spinal neurons including ChAT+ motor neurons in the ventral horn, with minimal overlap of white matter including astrocytes identified by GFAP+ staining (**Fig. 3, C to F, and fig. S4A**)(*32*, *33*, *75*). Flow cytometry enrichment of AAV9-GFP transduced cortical nuclei revealed 87±3.5% conversion of *SMN2* C6T (**Fig. 3G)**(*74*, *76*). Prior studies of AAV9 tropism demonstrated almost exclusive (≥90%) neuronal targeting in the cortex following neonatal ICV injection in mice(*72*), indicating that AAV9-ABE enables efficient base editing in transduced neurons *in vivo*, consistent with prior studies(*74*, *155*, *156*).

The composition of the cortex and high overall transduction efficiency allow for proper dissociation and relatively clean nuclear isolation of a large number of nuclei by flow cytometry that enables high-quality downstream sequencing analysis(*74*, *76*). We observed 87±3.5% base editing conversion *SMN2* C6T (**Fig. 3G**). Though AAV9-mediated transduction of spinal cells is lower(*21*), we performed flow cytometry enrichment of GFP+ nuclei among auto-fluorescent cell debris from dissociated spinal cord tissue and observed a 6.2-fold enrichment of edited cells, amounting to 45%±3.1% in the GFP-enriched population relative to bulk tissue 7.5%±0.5% (**Fig. S4B**). We furthermore observed an improvement in both CMAP and MUNE outcomes and a significant improvement in lifespan (**Fig. 4, A to C**). Since upregulation of SMN protein levels is required for rescue of electrophysiological deficits in spinal motor neurons and the survival of Δ7SMA mice(*59*, *73*, *88*, *117*, *157*), these data collectively confirm that ICV injection of AAV9-ABE enables efficient base editing conversion of *SMN2* C6T in transduced spinal motor neurons *in vivo*.

Normal SMN protein production is essential to the function, survival, and long-term health of all animals(*58*–*61*, *158*–*163*). The *SMN2* and *SMN1* genes differ only by C6T and their genomic loci share ≥99.9% sequence identity(*1*, *4*, *5*). Thus base editing conversion of C6T effectively converts native pathogenic *SMN2* genes to native *SMN1* equivalents while maintaining endogenous regulation that does not induce abnormal SMN transcript levels or protein production (**Fig. 2, F and G,** and **fig. S3G**)(*1*, *4*, *5*, *47*), thereby avoiding potential toxicities associated with either *SMN* overexpression or insufficiency in targeted tissues(*21*, *23*, *24*, *28*, *29*, *35*). Despite the unusually short window for rescue of Δ7SMA mice (≤6 days), AAV9-ABE improved lifespan from an average of 17 days to 23 days (**Fig. 4C, and fig. S5B**). This lifespan extension was also replicated in a separate Δ7SMA mouse colony in another lab (Burghes lab), despite differences in birthweight and lifespan between animals raised in the two facilities (birthweight two-tailed t-test *p<*0.01, lifespan Mantel-Cox test *p*=0.03, see **Materials and** **Methods** for more detail), where the average lifespan of untreated animals was 13 days (median 13 days, maximum 17 days) that increased to average of 17 days (median 18 days, maximum 20 days) in AAV9-ABE treated animals (Mantel-Cox test *p*=0.04, (**fig. S5, C and D**).

Upregulation of SMN protein levels improves motor function and life expectancy of SMA patients and animal models if achieved prior to onset of neuromuscular pathology and symptoms(*13*, *32*, *86*–*88*, *93*), prior to PND4-6 in ∆7SMA mice (**Fig. 4B**)(*32*, *88*, *89*, *91*–*93*). In cells, exon 7 splicing is not fully rescued until seven days post D10 transfection (**fig. S5A**), and *in vivo* base editing typically takes weeks to affect protein levels(*81*). AAV9-ABE mediated rescue of SMN protein levels thus surpasses the extremely short therapeutic window of Δ7SMA mice, and resembles *post-symptomatic* rescue of SMN protein levels in this model.

Transient SMA drug administration can ameliorate SMA pathology and extend survival of Δ7SMA mice(*73*, *97*, *98*). Repeated dosing of ~4-80 µg nusinersen can enable long-term rescue of ∆7SMA mice(*99*, *164*), while a single ICV injection of nusinersen at PND0 can extend survival of Δ7SMA mice by several weeks(*99*). In this study, we investigated whether extending the extremely short therapeutic window of Δ7SMA mice could improve AAV9-ABE mediated rescue and to this end we chose to co-administer a single low dose of nusinersen (1 µg) to attenuate disease progression for several days. This combination therapy approach allowed a greater opportunity for base editing rescue to take hold that more closely resembles *pre-symptomatic* Zolgensma administration at ≤PND3 (**Fig. 4H**). The therapeutic window for treatment of SMA patients ranges from months to years(*13*, *24*, *84*–*87*, *100*), which we anticipate would provide ample opportunity for base editing-mediated restoration of SMN protein levels to take place without the need for co-administration of a transient therapeutic.

**
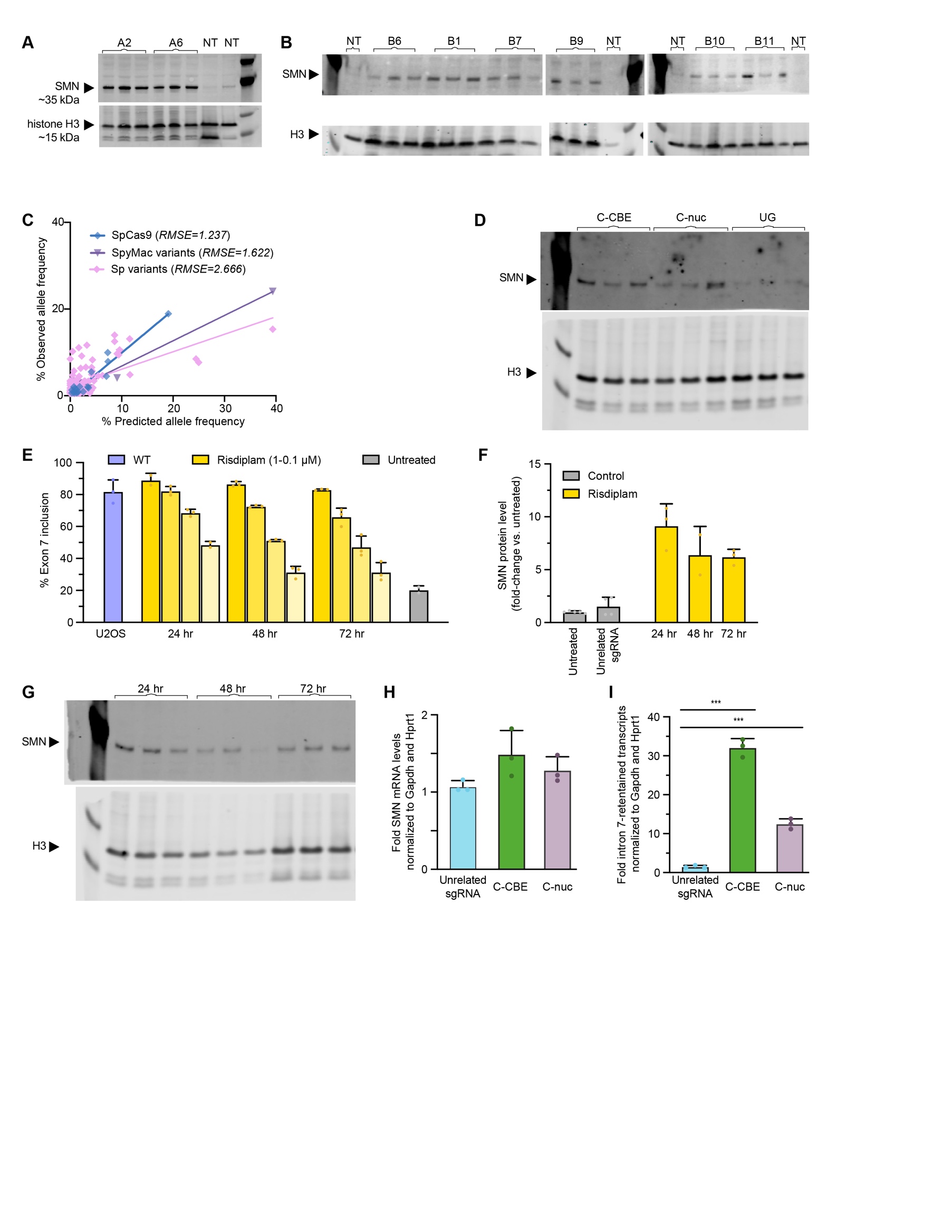
**

**Fig S1. (A)** Western blot accompanying Fig. 1D. **(B)** Western blot accompanying Fig. 1F **(C)** Correlation of inDelphi-predicted edited alleles with the observed frequency of edited alleles for either SpCas9, or SpCas9 engineered and evolved variants (SpCas9 family) and SpyMac family Cas components. **(D)** Western blot accompanying Fig. 1H. **(E)** Splicing time course of exon 7 inclusion in risdiplam-treated Δ7SMA mESCs compared to untreated Δ7SMA mESCs and wild-type human U2OS cells. The dark yellow to light yellow bars indicates risdiplam doses of 1.0, 0.5, 0.25, or 0.1 µM. **(F, G)** Bar graph and Western blot of SMN protein levels over time in Δ7SMA mESCs treated with risdiplam relative to untreated cells, normalized to histone H3. **(H)** Exon 7 mRNA transcript levels in Δ7SMA mESCs edited by C-CBE and C-nuc paired with exon 8 splice acceptor-targeting sgRNAs, normalized to Gapdh and Hprt1, relative to EA-BE4 base editor paired with an unrelated sgRNA control. **(I)** Fold-change of intron 7-retained transcripts in Δ7SMA mESCs edited by C-CBE and C-nuc compared to EA-BE4 base editor paired with an unrelated sgRNA control, normalized to Gapdh and Hprt1. ***≤0.005 by Welch’s two-tailed t-test. UG=unrelated guide; NT=no treatment.

**
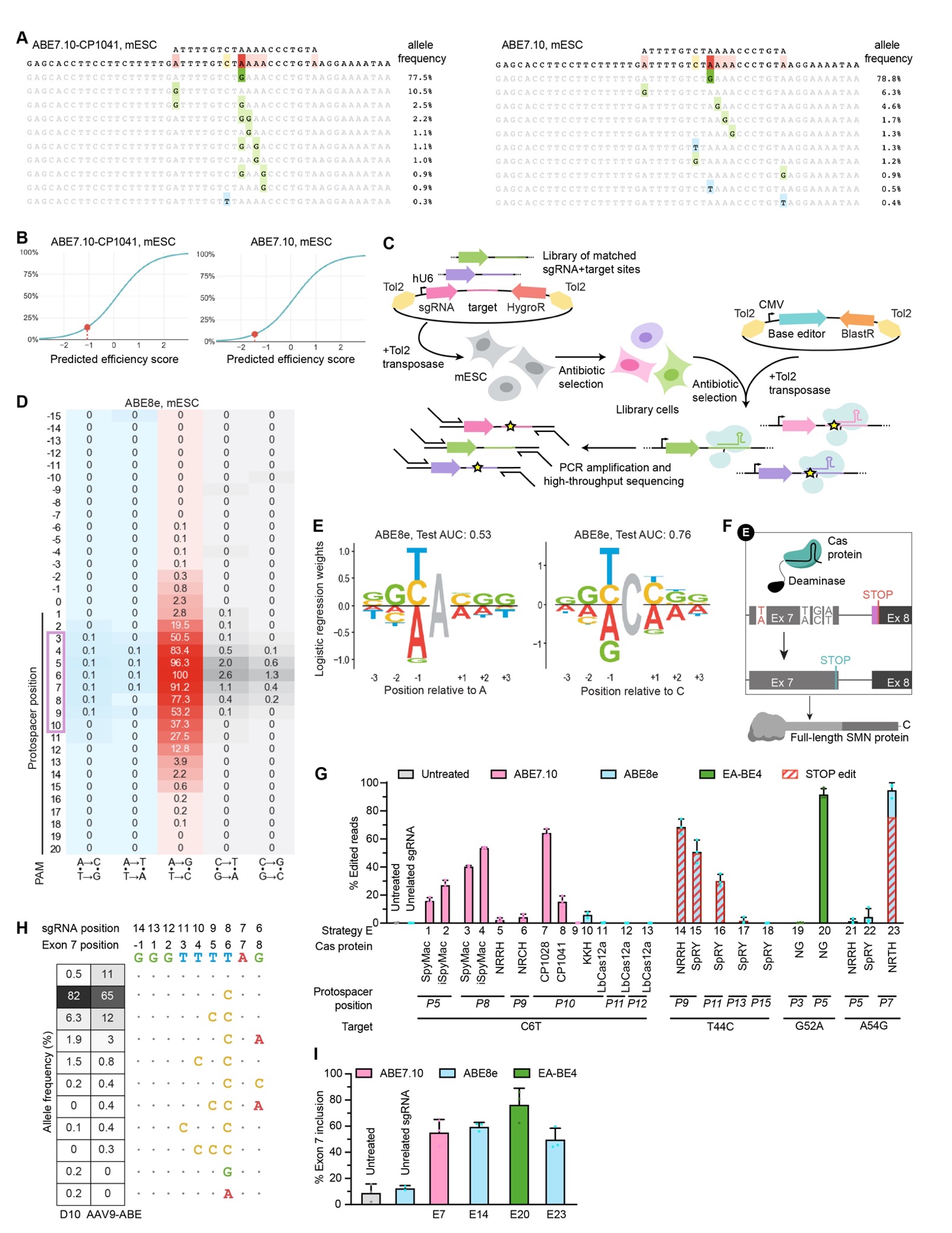
**

**Fig. S2. (A, B)** BE-Hive model predictions of ABE7.10-CP1041 and ABE7.10-SpCas9 base editing for *SMN2* C6T with the only available NGG-PAM sgRNA. **(A)** The relative frequency of the corresponding base editing outcomes. **(B)** The expected base editing efficiency for the indicated strategies in mESCs. **(C)** Illustration of the comprehensive context library, a high-throughput genome integrated library of sgRNA:target pairs to enable comprehensive characterization of ABE8e editing outcomes. **(D)** ABE8e activity profile. Values show the percent editing efficiency for each protospacer position (*P#*), for the base editing outcome that is specified at the bottom of each column, relative to the most efficiently edited position (*P6*). Red indicates canonical A-to-G base editing, blue indicates rare A-to-C and A-to-T, and gray indicates other rare mutations. Protospacer positions with values ≥30% of maximum are outlined in purple, indicating the ABE8e editing window. **(E)** Sequence motif for canonical A-to-G, and non-canonical C-to-T base editing by ABE8e from logistic regression modeling. The sign of each learned weight indicates a contribution above (positive sign) or below (negative sign) the mean activity. Logo opacity is proportional to the Pearson’s *r* on held-out sequence contexts. **(F)** Adenine base editing strategies targeting various splice regulatory elements (SREs) in exon 7 (strategy E). **(G)** Base editing efficiency in Δ7SMA mESCs of strategies E1-23 that target various SREs in exon 7. The target nucleotide position within the protospacer (*P#*) is indicated below. Red stripes indicate the fraction of alleles that ablate the exon 7 stop codon. **(H)** Edited allele frequencies following D10 transfection in Δ7SMA mESCs (*n=*3), or of GFP+ cortical nuclei from AAV9-ABE + AAV9-GFP treated Δ7SMA mice (*n=*5) normalized to untreated controls (*n=*3). All editable nucleotides at the target site are shown, edited alleles shown in this table occur at ≥ 0.2% of outcomes in one or more replicates. **(I)** Exon 7 inclusion in Δ7SMA mESCs edited by the indicated strategies, measured by automated electrophoresis.

**
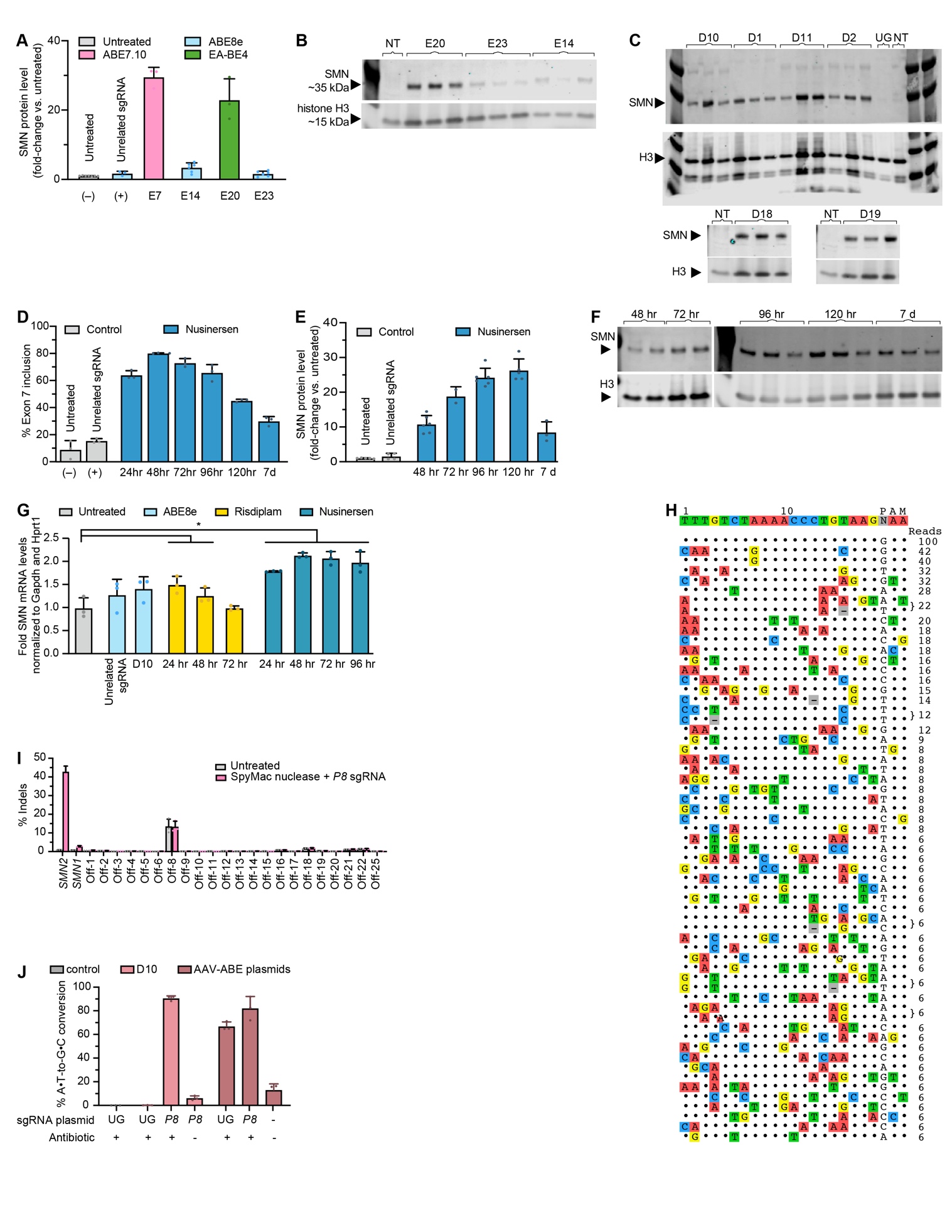
**

**Fig. S3. (A, B)** Bar graph and Western blot of SMN protein levels in Δ7SMA mESCs edited by the indicated strategies, normalized to histone H3. **(C)** Western blot accompanying Fig. 2G. **(D)** Time course of exon 7 inclusion in Δ7SMA mESCs treated with 20 µM nusinersen, measured by automated electrophoresis. **(E, F)** Bar graph and Western blot of SMN protein levels over time in Δ7SMA mESCs treated with nusinersen relative to untreated cells, normalized to histone H3. **(G)** Exon 7 mRNA transcript levels in Δ7SMA mESCs under the indicated conditions, *<0.05 by Welch’s two-tailed t-test. **(H)** CIRCLE-Seq nominations of candidate off-target sites in HEK293T cell human genomic DNA treated *in vitro* with purified SpyMac nuclease protein and *P8* sgRNA. Mismatches at each off-target locus are shown compared to the on-target sequence in the top row. **(I)** On-target and off-target indel frequency of Spy-mac nuclease and *P8* sgRNA in HEK293T cells. **(J)** ABE-mediated editing of *SMN2* C6T by strategy D10 transfection conditions compared to transfection with the dual AAV9-ABE plasmids that encode split-intein ABE8e-SpyMac and the *P8* sgRNA. Controls of untreated cells (NT) and treatment with ABE8e-SpyMac+unrelated sgRNA (UG) are shown. ‘sgRNA’ indicates co-transfection with a Tol2-sgRNA plasmid that allows for hygromycin antibiotic enrichment of transfected cells, ‘antibiotic’ indicates whether hygromycin selection was performed. UG=unrelated guide; NT=no treatment.

**
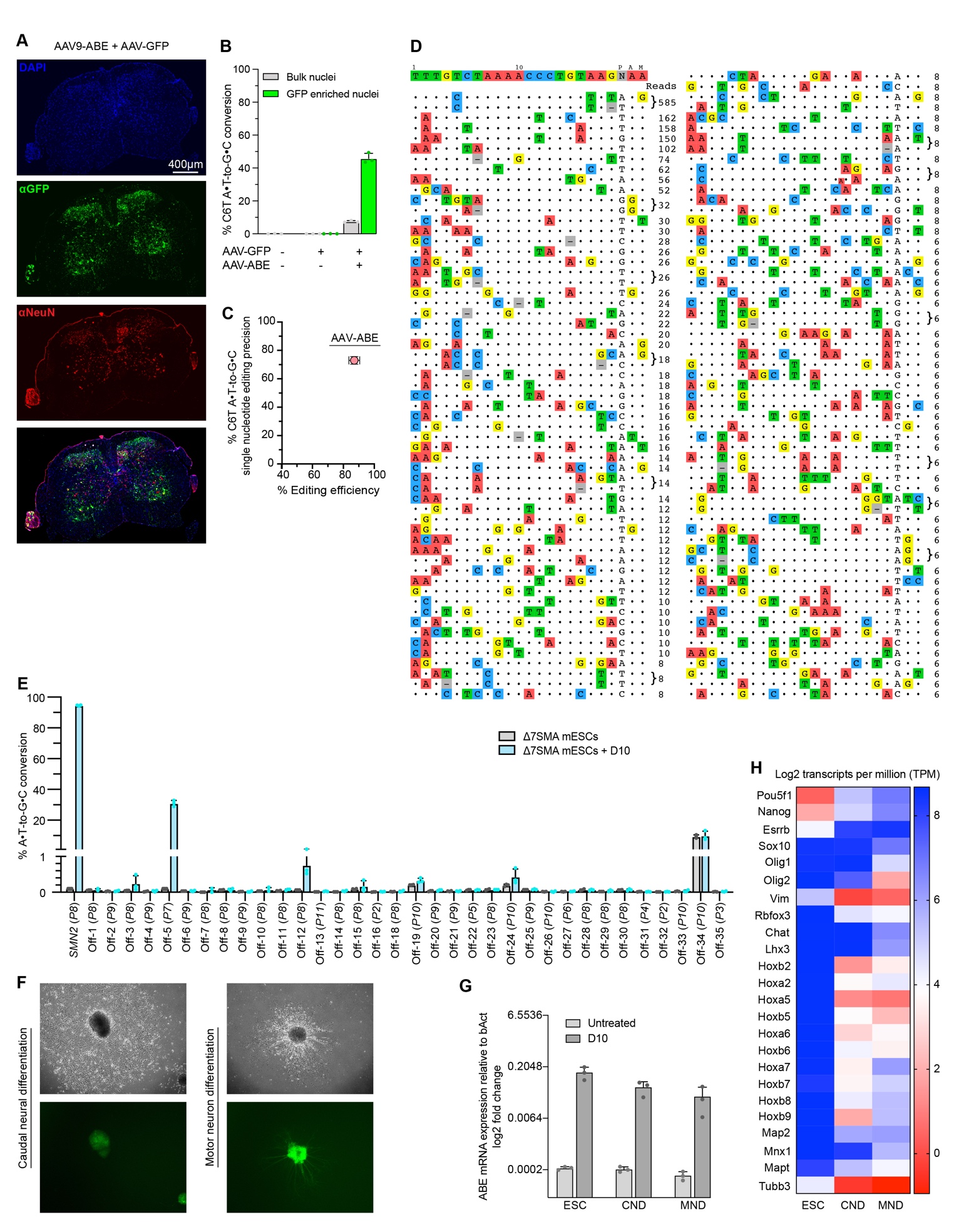
**

**Fig. S4. (A)** Immunofluorescence images of spinal cord sections from wild-type Δ7SMA mice at 25 weeks that received AAV9-ABE + AAV9-GFP in a 10:1 ratio by neonatal ICV injection, stained for GFP to indicate AAV transduction, NeuN as a marker of post-mitotic neurons, and DAPI to stain all nuclei. **(B)** *In vivo* base editing conversion of C6T in the spinal cord of Δ7SMA mice treated with AAV9-ABE + AAV9-GFP in bulk dissociated tissue, and GFP+ enriched nuclei. **(C)** CIRCLE-Seq nominations of candidate off-target sites in NIH3T3 cell genomic DNA treated *in vitro* with purified Spy-mac nuclease and *P8* sgRNA. Mismatches at each off-target locus are shown relative to the sgRNA above. **(D)** On-target and off-target base editing of strategy D10 in Δ7SMA mESCs. Bars show editing of the highest edited nucleotide (*P#* shown in parenthesis) at each locus. **(E)** Fluorescence imaging of CND and MND differentiated Δ7SMAmESCs that harbor the Mnx1:GFP reporter of motor neurons and stably integrated with the D10 ABE strategy. **(F)** RT-qPCR for ABE8e expression in Δ7SMAmESCs (*n*=3) and differentiated MND (*n*=3) and CND (*n*=3) populations, previously transfected with the D10 strategy. **(G)** Gene expression analysis of Δ7SMAmESCs (*n*=3), and CND (*n*=3) and MND (*n*=3) differentiated cells showing expression levels of various motor neuron specific, neuron specific, spinal cord patterning, glia, and embryonic stem cell markers.

**
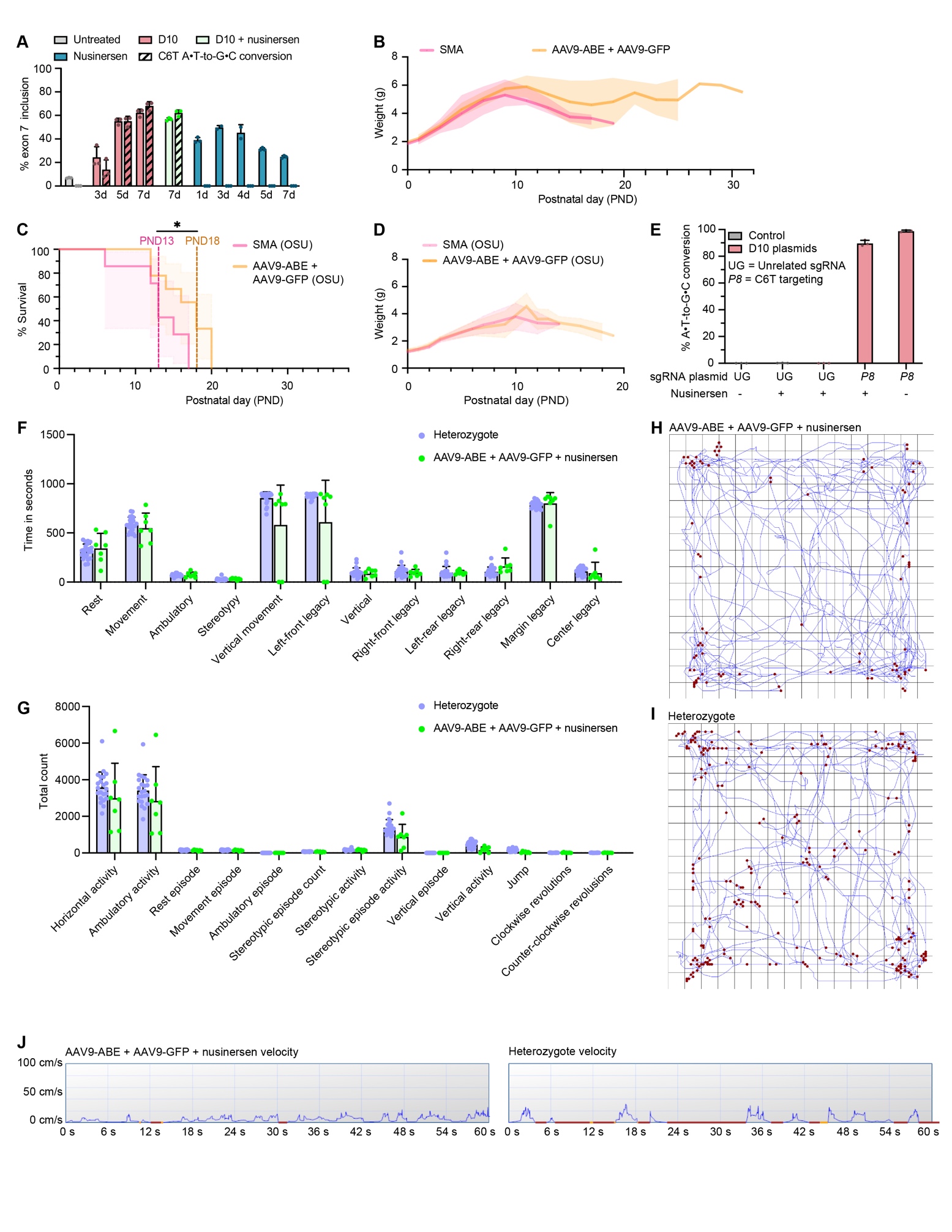
**

**Fig. S5. (A)** Time course exon 7 inclusion in Δ7SMA mESCs treated with the D10 strategy and 20 µM nusinersen as indicated, showing the frequency of U-to-C conversion at C6T in spliced transcripts, measured by high-throughput sequencing of reverse transcribed *SMN* mRNA. **(B)** Body weight measurements for the indicated Δ7SMA mouse cohorts at the Broad Institute, as shown in **Fig. 4C**. **(C, D)** Body weight measurements and Kaplan-Meier curve of Δ7SMA neonates at Ohio State University (OSU) treated with AAV9-ABE (*n*=9), compared to uninjected controls (*n*=9). The asterisk indicates *p*=0.04 by Mantel-Cox test. Error bars and graph line shading in bodyweight measurements represent standard deviations of ≥3 independent biological replicates. Graph line shading in survival curve represents 95% CI.

**(E)** ABE-mediated editing of *SMN2* C6T by strategy D10 in Δ7SMA mESCs with, and without the addition of 20 µM nusinersen. UG=unrelated guide. **(F, G)** Voluntary movement by open field tracking at PND40 for 15 min of Δ7SMA mice treated with AAV9-ABE+nusinersen (*n*=7) compared to wild-type controls (*n*=22). Behaviors did not differ significantly from wild-type (Mann-Whitney test *p>0.5*). Uninjected and nusinersen-only injected Δ7SMA control animals were not available due to their short lifespan. Graphs show (F) the amount of time in seconds spent on the indicated activity, and (G) the total counts of a given behavior over the measured period. **(H-J)** Trace (H-I) and velocity (J) plots of PND40 Δ7SMA mice treated with AAV9-ABE+nusinersen, or healthy heterozygous control mice in the open field test.

**Table S1.** Strategy A rescue haplotypes.

| **A2** | TAAATTAAGGAGTAAGTCTGCCAG-----------TGAAT |
| --- | --- |
| **A2** | TAAATTAAGGAGTAAGTCTGCCAG--------AAGTGAAT |
| **A2** | TAAATTAAGGAGTAAGTCTGCCAGCATTA---------AT |
| **A2** | TAAATTAAGGAGTAAGTCTGCCAGCAGTTT---------- |
| **A2** | TAAATTAAGGAGTAAGTCTGCCAGCAT-----AAGTGAAT |
| **A2** | TAAATTAAGGAGTAAGTCT--------------------- |
| **A2** | TAAATTAAGGAGTAAGTCTGCCAGCATTATGAA------T |
| **A2** | TAAATTAAGGAGTAAGTCTGCCAGCATTA---------AT |
| **A2** | TAAATTAAGGAGTAAGTCTGCCAGCA-----AAAGTGAAT |
| **A2** | TAAATTAAGGAGTAAGTCT--------------------- |
| **A2** | TAAATTAAGGAGTAAGTCTGCCAGCATTATG-----GAAT |
| **A2** | TAAATTAAGGAGTAAGTCTGCCAGCAT------------- |
|  |  |
| **A3** | TAAATTAAGGAGTAAGTCTGCCAGCATTATGAA------T |
| **A3** | TAAATTAAGGAGTAAGTCTGCCAG-----------TGAAT |
| **A3** | TAAATTAAGGAGTAAGTCTGCCAGCATTATG----TGAAT |
|  |  |
| **A5** | TAAATTAAGGAGTAAGTCTGCCAGCATTATGAA------T |
| **A5** | TAAATTAAGGAGTAAGTCTGCCAGCATTAT---------- |
| **A5** | TAAATTAAGGAGTAAGTCTGCCAGCATTA-----GTGAAT |
| **A5** | TAAATTAAGGAGTAAGTCTGCCAGCA------AAGTGAAT |
| **A5** | TAAATTAAGGAGTAAGTCTGCCAG-----------TGAAT |
|  |  |
| **A6** | TAAATTAAGGAGTAAGTCTGCCAGCATTATGAA------T |
|  |  |
| **A14** | TAAATTAAGGAGTAAGTCTGCCAGCATTA----AGTGAAT |

Rescue haplotypes, as defined in the main text, included here occur in ≥5% of reads in one or more replicates.

**Table S2.** Strategy B rescue haplotypes.

| **B1** | CATTTGCAGGAAAATGCTGGCATAGAGCAGCACTAAATGA | Terminal amino acids |
| --- | --- | --- |
| **B1** | CATTTGCAGGAAATTGCTGGCATAGAGCAGCACTAAATGA | Terminal amino acids |
| **B1** | TCATTTGC---------TGGCATAGAGCAGCACTAAATGA | Alternative splicing |
| **B1** | TCATTTGCA---AATGCTGGCATAGAGCAGCACTAAATGA | Alternative splicing |
| **B1** | CATTTGCAGGAAAGTGCTGGCATAGAGCAGCACTAAATGA | Terminal amino acids |
| **B1** | TCAT--------------GGCATAGAGCAGCACTAAATGA | Alternative splicing |
| **B1** | TC------------TGCTGGCATAGAGCAGCACTAAATGA | Alternative splicing |
| **B1** | TCATTTGCAGGA----------TAGAGCAGCACTAAATGA | Terminal amino acids |
|  | | |
| **B6** | TCATTTGCAGGAAATGC----ATAGAGCAGCACTAAATGA | Terminal amino acids |
| **B6** | TCATTTGCAGGAAATGCTG-CATAGAGCAGCACTAAATGA | Terminal amino acids |
| **B6** | TCATTTGCAGGAAATGCTGG-ATAGAGCAGCACTAAATGA | Terminal amino acids |
| **B6** | TCATTTGCAGGAAATGCTGG-TGAGAGCAGCACTAAATGA | Terminal amino acids |
| **B6** | TCATTTGCAGGAAATGCTGG---AGAGCAGCACTAAATGA | Terminal amino acids |
| **B6** | TCATTTGCAGGAAATGCTGG--TAGAGCAGCACTAAATGA | Terminal amino acids |
|  | | |
| **B7** | TCATTTGCAGGAAAT--------AGAGCAGCACTAAATGA | Terminal amino acids |
| **B7** | TCATTTGCAGGAAATGCTGG-----AGCAGCACTAAATGA | Terminal amino acids |
| **B7** | TCATTTGCAGGAAATGCTGG-ATAGAGCAGCACTAAATGA | Terminal amino acids |
| **B7** | TCATTTGCAGGAAATGCTG-CATAGAGCAGCACTAAATGA | Terminal amino acids |
| **B7** | TCATTTGCAGGAAATGCTG--------CAGCACTAAATGA | Terminal amino acids |
| **B7** | TCATTTGCAGGAAATGCTGG--TAGAGCAGCACTAAATGA | Terminal amino acids |
|  | | |
| **B9** | TCATTTGCAGGAAATGCTGGCCATAGAGCAGCACTAAATG | Terminal amino acids |
| **B9** | TCATTTGCAGGAAATGCTGG----------CACTAAATGA | Terminal amino acids |
| **B9** | TCATTTGCAGGAAATGCAAATTTCCTGAGCAGCACTAAAT | Terminal amino acids |
| **B9** | TCATTTGCAGGAAATGCTGG-----AGCAGCACTAAATGA | Terminal amino acids |
| **B9** | TCATTTGCAGGAAATGCTG--ATAGAGCAGCACTAAATGA | Terminal amino acids |
| **B9** | TCATTTGCAGGAAATGC---CATAGAGCAGCACTAAATGA | Terminal amino acids |
| **B9** | TCATTTGCAGGAAATGCTGG---AGAAGCAGCACTAAATG | Terminal amino acids |
| **B9** | TCATTTGCAGGAAATGCTG------AGCAGCACTAAATGA | Terminal amino acids |
| **B9** | TCATTTGCAGGAAATGCTGGCA--GAGCAGCACTAAATGA | Terminal amino acids |
| **B9** | TCATTTGCAGGAAATGCTGG---GGAGCAGCACTAAATGA | Terminal amino acids |
|  | | |
| **B10** | TCATTTGCAGGAAATGCTGGCAATAGAGCAGCACTAAATG | Terminal amino acids |
| **B10** | TCATTTGCAGGAAATGCTGG----------CACTAAATGA | Terminal amino acids |
| **B10** | TCATTTGCAGGAAATGCTGGCA--GAGCAGCACTAAATGA | Terminal amino acids |
| **B10** | TCATTTGCAGGAAATGCTGG-------CAGCACTAAATGA | Terminal amino acids |
| **B10** | TCATTTGCAGGAAATGCT-----AGAGCAGCACTAAATGA | Terminal amino acids |
|  | | |
| **B11** | TCATTTGCAGGAAATGCTGGCAT------GCACTAAATGA | Terminal amino acids |
| **B11** | TCATTTGCAGGAAATGCTGG-------CAGCATTAAATGA | Terminal amino acids |
| **B11** | TCATTTGCAGGAAATGCTGGAAATGTCATTTAGAGCAGCA | Terminal amino acids |
| **B11** | TCATTTGCAGGAAATGCTGGCAATAGAGCAGCACTAAATG | Terminal amino acids |
| **B11** | TCATTTGCAGGAAA----------GAGCAGCACTAAATGA | Terminal amino acids |

Rescue haplotypes, as defined in the main text, included here occur in ≥5% of reads in one or more replicates. The mechanism of SMN protein rescue, by disrupting splicing or disrupting the SMNΔ7 terminal degron, is indicated.

**Table S3.** Oligonucleotides used in this study.

| **Strategy A** | **sgRNA** | **Scaffold** |
| --- | --- | --- |
| **1** | G AGTCTGCCAGCATTATGAAA | SpCas9 |
| **2-4** | GTCTGCCAGCATTATGAAAG | SpCas9 |
| **5-7** | G TCTGCCAGCATTATGAAAGT | SpCas9 |
| **8-9** | G CTGCCAGCATTATGAAAGTG | SpCas9 |
| **10** | G TGCCAGCATTATGAAAGTGA | SpCas9 |
| **11-12** | G AAAGTAAGATTCACTTTCAT | SpCas9 |
| **13-15** | G AAAAGTAAGATTCACTTTCA | SpCas9 |
| **16** | G CAAAAGTAAGATTCACTTTC | SpCas9 |
| **17-18** | G ACAAAAGTAAGATTCACTTT | SpCas9 |
| **19** | G TACAAAAGTAAGATTCACTT | SpCas9 |
|  | | |
| **Strategy B** | **sgRNA** | **Scaffold** |
| **1** | G TTCTCATTTGCAGGAAATGC | SpCas9 |
| **2-3** | G TCTCATTTGCAGGAAATGCT | SpCas9 |
| **4-5** | G ATTTGCAGGAAATGCTGGCA | SpCas9 |
| **6-7** | G TTTGCAGGAAATGCTGGCAT | SpCas9 |
| **8-9** | G TTGCAGGAAATGCTGGCATA | SpCas9 |
| **10-11** | G TGCAGGAAATGCTGGCATAG | SpCas9 |
| **12** | G CAGGAAATGCTGGCATAGAG | SpCas9 |
| **13-14** | G ATTTAGTGCTGCTCTATGCC | SpCas9 |
| **15-16** | G CATTTAGTGCTGCTCTATGC | SpCas9 |
|  | | |
| **Strategy C** | **sgRNA** | **Scaffold** |
| **C-CBE** | G TTTCctgcaaatgagaaatt | SpCas9 (iSpyMac PAM=NAA) |
| **C-nuc** | GCTCTATGCCAGCATTTCctg | SpCas9 (EA-BE4-NG PAM=NG) |
|  | | |
| **Strategy D** | **sgRNA** | **Scaffold** |
| **1-3** | G GTCTAAAACCctgtaaggaa | SpCas9 |
| **4-6** | G TGTCTAAAACCctgtaagga | SpCas9 |
| **7-9** | G TTGTCTAAAACCctgtaagg | SpCas9 |
| **10-13** | G TTTGTCTAAAACCctgtaag | SpCas9 |
| **14-16** | G TTTTGTCTAAAACCctgtaa | SpCas9 |
| **17-19** | GATTTTGTCTAAAACCctgta | SpCas9 |
|  | | |
| **Strategy E** | **sgRNA** | **Scaffold** |
| **1-2** | G GTCTAAAACCctgtaaggaa | SpCas9 |
| **3-5** | G TTTGTCTAAAACCctgtaag | SpCas9 |
| **6** | G TTTTGTCTAAAACCctgtaa | SpCas9 |
| **7-8** | GATTTTGTCTAAAACCctgta | SpCas9 |
| **9-10** | GATTTTGTCTAAAACCctgtaag | SaCas9 |
| **11** | G ATTTTGTCTAAAACCCTG | LbCas12a |
| **12** | GATTTTGTCTAAAACCCT | LbCas12a |
| **13** | G TGATTTTGTCTAAAACCC | LbCas12a |
| **14-15** | G CTTAATTTAAGGAATGTGAG | SpCas9 |
| **16** | G TCCTTAATTTAAGGAATGTG | SpCas9 |
| **17** | G ACTCCTTAATTTAAGGAATG | SpCas9 |
| **18** | G TTACTCCTTAATTTAAGGAA | SpCas9 |
| **19** | G TCCTTAATTTAAGGAATGTG | SpCas9 |
| **20-21** | G ACTCCTTAATTTAAGGAATG | SpCas9 |
| **21-22** | G AAGGAGTAAGTCTGCCAGCA | SpCas9 |
| **23** | G TTAAGGAGTAAGTCTGCCAG | SpCas9 |
|  | | |
| **Primers** | | |
| **gDNA locus specific primer sequences for PCR1** | **Forward** | **Reverse** |
| SMN exon 7 + ISSN1 | TCAACTTTCTAACATCTGAACTTTTT | TGTCTTGTGAAACAAAATGCTT |
| SMN exon 8 | GAAGAAATGAGGCCAGTTATCG | GTCCACAGAGGACATGGTTT |
|  | | |
| **Human off-target analysis locus specific primers for PCR1** | **Forward** | **Reverse** |
| ***SMN2*** | GCTATCTATATCTATATAGCTAT | CCTTTCAACTTTCTAACATCTG |
| ***SMN1*** | GCTATCTATGTCTATATAGCTAT | CCTTTCAACTTTTTAACATCTG |
| **off-1** | CAATGTGGGTACAGGCATTGGAT | CCTTGCACTGGATTCAAGACAGGT |
| **off-2** | ATGAGGTATCCACCGAATGTCCA | ATGCATTAACCAATTAGGCTGCAT |
| **off-3** | ATATGTGGCCTGGCTTTGTTGTT | CCCTGGTCACAAAGGTAATAAGAGGA |
| **off-4** | TGTTATGCATCAAAAATTGCTTA | TGGATGTGTGAATATTCTTCTTGACG |
| **off-5** | AGTGGATGTGTATGGTGCCAAAT | TCAATTACCTCTGTTTAAAATGCTCT |
| **off-6** | GCACAGTGTTTTGGGCATCTTGATA | TCTGCTTCAAATCTTGGGGAAAA |
| **off-8** | TCGGCAAACCAAGAGATTGTGAC | TGTGTGAATTACTCCTTCTCCATTTC |
| **off-9** | AGAGACGGGTTTCACCATG | AGGGCGGGGTAATAATGAGTTAG |
| **off-10** | CTCGTGTGCAGTGATGTGATGTC | CCTTGATGGGCAGCATAAAGTATGT |
| **off-11** | TTTCTGTGAAAATCCACAGTGAC | AAGCTAAGAAGTTTGAAATTATCCCT |
| **off-12** | AAAGAATGGTTTCTTTTATTTGCTA | GGATATTAAGCTTTGATGTTTAG |
| **off-13** | CAAAAAGACGTTACTTAATGTTGACCA | TCAGCCTTGGCATTTTCATACACA |
| **off-14** | CCTTGAAGATACAACCGTGTGAG | TGAGAAGAATAAAATAGATCTGTGGT |
| **off-15** | AGAGCTGCTGAGAGGACTGAC | TGATGTAAGACAGGCATAGAATAGACA |
| **off-16** | TCAAGCGATTTGATGCCAACAGTA | CATGAAAGGAAGAAACCCCGTTT |
| **off-17** | CAGAGCCTCCAAAACCTGACAAA | AAGAAGCTCTGTGTATAGCCATCAT |
| **off-18** | ATTTGTCAATTTTGTGTTTTGTT | TTCAATAAATGGTGGGAAAAGTGG |
| **off-19** | TTGATTTTGTATCCCACAGCTTC | AAACAAGCCTACCACCTCTCACCA |
| **off-20** | CTGCAAGCTGCACTCACTGATGT | CTGGCTGCCACTCCCACCT |
| **off-21** | AAGGTGAGCTGAAGTCTGAAGTT | GAGATTGTGCCACAGTACCACAGC |
| **off-22** | CTGTTGAGGTGGTTGTCAGGGAA | GGAGCTGTTTCCTCCGCTAGTCAG |
| **off-25** | CACAGAAATCTTCCCACCAAACA | AGAGCACAGTGCCTGTGTCATTTC |
|  | | |
| **Mouse off-target analysis locus specific primers for PCR1** | **Forward** | **Reverse** |
| ***SMN2*** | TCAACTTTCTAACATCTGAACTTTTT | TGTCTTGTGAAACAAAATGCTT |
| **off-1** | GGAGCACCCTCACAGAAGCA | GCCTTTCCCATAAGGAACAGCA |
| **off-2** | GCTGCCATGTGGTTTCTGTG | AAAGGCCACATCACGAATTCTA |
| **off-3** | GCCATCTCACCAGCCTCCAT | GGGATGGGTACCTGAGCCTTA |
| **off-4** | CCAGGAAGATGCTGGCAAGA | GATGGTTGGGGGAGGGTGTA |
| **off-5** | ATTGATGGGGAAGGCAGCAT | CCTTTATCAGCCTTTATTCAGCCAAC |
| **off-6** | GCCTAGCCACCCAAGTGTCC | CCACCCCCAACAACAACAAA |
| **off-7** | AGCCTGTGGCTGGGAAGAAG | GCCTTGAAGCATTAGCTCCAGA |
| **off-8** | GGCCATTTTCTGATATGTCCTTCC | TGCCCATCAAGTATTCTGAAGCA |
| **off-9** | ATCCCAGCTGCTGTGCATGT | CATTGTCTGCTGCCCACTCC |
| **off-10** | GGGCCTCCCAATAATGCTTC | GTGCCTGTTTGCTTGCTGCT |
| **off-11** | CAGCAAATGAATTATGGCTCACA | TGATAGGACCACATTAGATACTGTTCA |
| **off-12** | GGAGTCCGGGATCCAAGATG | AGGCATTCCACCACACATGG |
| **off-13** | CAGAGCAGGGCTTTCTGTGC | TTCCCAGTTTTGAAGCAACTCG |
| **off-14** | CCTCACTGGAAATGGCATCG | GGGAGGCCACTCATTTTTGTG |
| **off-15** | CCTGTGTGTGGGCTTGCTCT | ATCCTGGGTGTGCAATGTGG |
| **off-16** | AAGCATGGCAAAGGGAAGAA | CTCGGGGATGAAGGAGAAGC |
| **off-18** | TTGGTCCAGGGAAGCCATTT | CGCATAGCTTGCACTTGTGG |
| **off-19** | TCATTTCGAAGCAGTGTTTTTCA | TGTCCATAAGGCACATGTTTTGC |
| **off-20** | TGCTGAGATATCACCCAGGAC | TGTTTCTGAGTTCAAGTCCACTG |
| **off-21** | AGCCATCTCTCCAGCCCTGA | CTGCTGGTCTCTGCCGTTGT |
| **off-22** | CAAGGCTGTAGGAATGCAATGG | AAAGCCAGGGAAATGGCAGA |
| **off-23** | TTCCCAGATGGAAAGGCTCA | TGCCTTGGTCATGGTGTCTG |
| **off-24** | CCCACTCCAGTGAACGGACA | AATCGCTTGGTGGGTGCTCT |
| **off-25** | CCTAGCAGTGGGGAAGCTGT | TGGACCTGGATTTTGTCAGC |
| **off-26** | TGGGATGTGTTCACTAAGAACATTTTG | TTGGAGTTGTGTGGCCATGT |
| **off-27** | CCGAGGCACCACATTCTGA | TCAACTGCTGGCCAGGAAAA |
| **off-28** | CCACAACCACAGCCCACTTC | CTCAGCCATTGGGAGACAGG |
| **off-29** | AAGGCATTCATTGGCTTTTGA | TCCCTGGTCCATTCCATGTG |
| **off-30** | GAAGGAAAGGAAGGATAAAGGATT | AGCACCAGTACAGGGGAATG |
| **off-31** | AAGGCATTCATTGGCTTTTGA | TCCCTGGTCCATTCCATGTG |
| **off-32** | CATTGCAGCAGAGTTGTTGTG | GGGGAGCATCATGCAATACTGA |
| **off-33** | CATCCCTTCGAGGACGTGAA | GAGTGAGGCCCTTTCCCAGA |
| **off-34** | GACCAGCACCATTTGTTGAAGA | TCACTGTGACTCAAGCTGAACAA |
| **off-35** | GGTGTGGCCTTCAGACATGC | CCCCTTGGTCTGCTCGGACT |
|  | | |
| **SMN cDNA amplification primers** | **Forward** | **Reverse** |
| SMN exon 6 - exon 8 | CCCACCACCTCCCATATGTCCAG | ttttctcaactgcctcacca |
| SMN exon 4 - exon 6 | ACATCAAGCCCAAATCTGCTCCA | TCCCAAAGCATCAGCATCATCAA |
| Gapdh | TTGATGGCAACAATCTCCAC | CGTCCCGTAGACAAAATGGT |
| Hprt1 | CTGGTGAAAAGGACCTCTCGAAG | CCAGTTTCACTAATGACACAAACG |
|  | | |
| **SMN RT and cDNA locus specific primers for PCR1** | **Forward** | **Reverse** |
| oligo-dT with Read2 stub for RT |  | TTTTTTT GTGACTGGAGTTCAGACGTGTGCTCTTCCGATCT TTTTTTTTTTTTTTTTTTTTTTTTTTTTTT |
| Exon 6 - polyA | CCACCACCTCCCATATGTCCAGATT | GTGACTGGAGTTCAGACGTGTGCTCTTCCGATCT |
|  | | |
| **ABE8e RT-qPCR quantitation primers** | **Forward** | **Reverse** |
| ABE8e_qPCR | GCCGGCGCCATGATCCACTCTAG | TGTGCCAGGTGTCTCAGAGCCAGA |
| Actb | CTGGCACCACACCTTCTACA | CGTACATGGCTGGGGTGTTGAA |
|  | | |
| **SMA Δ7 mouse genotyping** | **Forward** | **Reverse** |
| mutant allele | CTC CGG GAT ATT GGG ATT G | GGT AAC GCC AGG GTT TTC C |
| wild-type allele | CTC CGG GAT ATT GGG ATT G | TTT CTT CTG GCT GTG CCT TT |
|  | | |
| **Base editing library amplification primers** | **Forward** | **Reverse** |
| Integrated read 1 stub - integrated U6 promoter | ACACTCTTTCCCTACACGAC | GTGGAAAGGACGAAACACCG |
|  | | |
| **Smart-seq2 RNA-sequencing primers** |  |  |
| Template switching oligo (TSO) | AGCAGTGGTATCAACGCAGAGTACrGrG+G | |
| 3' RT primer | AAGCAGTGGTATCAACGCAGAGTAC(T30)VN | |
| ISPCR primer | AAGCAGTGGTATCAACGCAGAGT | |

**References**

133. M. S. Cobb, F. F. Rose, H. Rindt, J. J. Glascock, M. Shababi, M. R. Miller, E. Y. Osman, P. F. Yen, M. L. Garcia, B. R. Martin, M. J. Wetz, C. Mazzasette, Z. Feng, C. P. Ko, C. L. Lorson, Development and characterization of an SMN2-based intermediate mouse model of spinal muscular atrophy. *Hum. Mol. Genet.* (2013), doi:10.1093/hmg/ddt037.

134. F. Farooq, S. Balabanian, X. Liu, M. Holcik, A. MacKenzie, p38 Mitogen-activated protein kinase stabilizes SMN mRNA through RNA binding protein HuR. *Hum. Mol. Genet.* **18**, 4035–4045 (2009).

135. R. N. Singh, N. N. Singh, "Mechanism of splicing regulation of spinal muscular atrophy genes" in *Advances in Neurobiology* (2018; file:///Users/marbab/Library/Application Support/Mendeley Desktop/Downloaded/Singh, Singh - Unknown - Mechanism of Splicing Regulation of Spinal Muscular Atrophy Genes.pdf), vol. 20, pp. 31–61.

136. S. Yoshimoto, N. I. F. Harahap, Y. Hamamura, M. Ar Rochmah, A. Shima, N. Morisada, M. Shinohara, T. Saito, K. Saito, P. S. Lai, M. Matsuo, H. Awano, I. Morioka, K. Iijima, H. Nishio, Alternative splicing of a cryptic exon embedded in intron 6 of SMN1 and SMN2. *Hum. Genome Var.* **3**, 1–3 (2016).

137. J. Seo, N. N. Singh, E. W. Ottesen, B. M. Lee, R. N. Singh, A novel human-specific splice isoform alters the critical C-terminus of Survival Motor Neuron protein. *Sci. Rep.* **6**, 1–14 (2016).

138. E. W. Ottesen, D. Luo, J. Seo, N. N. Singh, R. N. Singh, Human Survival Motor Neuron genes generate a vast repertoire of circular RNAs. *Nucleic Acids Res.* **47**, 2884–2905 (2019).

139. F. Allen, L. Crepaldi, C. Alsinet, A. J. Strong, V. Kleshchevnikov, P. De Angeli, P. Páleníková, A. Khodak, V. Kiselev, M. Kosicki, A. R. Bassett, H. Harding, Y. Galanty, F. Muñoz-Martínez, E. Metzakopian, S. P. Jackson, L. Parts, Predicting the mutations generated by repair of Cas9-induced double-strand breaks. *Nat. Biotechnol.* **37**, 64–82 (2019).

140. H. K. Kim, S. Min, M. Song, S. Jung, J. W. Choi, Y. Kim, S. Lee, S. Yoon, H. Kim, Deep learning improves prediction of CRISPR-Cpf1 guide RNA activity. *Nat. Biotechnol.* **36**, 239–241 (2018).

141. H. K. Kim, Y. Kim, S. Lee, S. Min, J. Y. Bae, J. W. Choi, J. Park, D. Jung, S. Yoon, H. H. Kim, SpCas9 activity prediction by DeepSpCas9, a deep learning–based model with high generalization performance. *Sci. Adv.* **5**, eaax9249–eaax9249 (2019).

142. J. G. Doench, N. Fusi, M. Sullender, M. Hegde, E. W. Vaimberg, K. F. Donovan, I. Smith, Z. Tothova, C. Wilen, R. Orchard, H. W. Virgin, J. Listgarten, D. E. Root, Optimized sgRNA design to maximize activity and minimize off-target effects of CRISPR-Cas9. *Nat Biotechnol*. **34**, 184–191 (2016).

143. J. H. Hu, S. M. Miller, M. H. Geurts, W. Tang, L. Chen, N. Sun, C. M. Zeina, X. Gao, H. A. Rees, Z. Lin, D. R. Liu, Evolved Cas9 variants with broad PAM compatibility and high DNA specificity. *Nature*. **556**, 57 (2018).

144. P. Chatterjee, J. Lee, L. Nip, S. R. T. T. Koseki, E. Tysinger, E. J. Sontheimer, J. M. Jacobson, N. Jakimo, A Cas9 with PAM recognition for adenine dinucleotides. *Nat. Commun.* **11**, 1–6 (2020).

145. X. Lin, H. Chen, Y. Q. Lu, S. Hong, X. Hu, Y. Gao, L. L. Lai, J. J. Li, Z. Wang, W. Ying, L. Ma, N. Wang, E. Zuo, H. Yang, W. J. Chen, Base editing-mediated splicing correction therapy for spinal muscular atrophy. *Cell Res.* **30** (2020), pp. 548–550.

146. A. Lapinaite, G. J. Knott, C. M. Palumbo, E. Lin-Shiao, M. F. Richter, K. T. Zhao, P. A. Beal, D. R. Liu, J. A. Doudna, DNA capture by a CRISPR-Cas9-guided adenine base editor. *Science (80-. ).* **369**, 566–571 (2020).

147. S. I. Nagaoka, F. Nakaki, H. Miyauchi, Y. Nosaka, H. Ohta, Y. Yabuta, K. Kurimoto, K. Hayashi, T. Nakamura, T. Yamamoto, M. Saitou, ZGLP1 is a determinant for the oogenic fate in mice. *Science (80-. ).* **367** (2020), doi:10.1126/science.aay5947.

148. H. K. Lee, H. E. Smith, C. Liu, M. Willi, L. Hennighausen, Cytosine base editor 4 but not adenine base editor generates off-target mutations in mouse embryos. *Commun. Biol.* **3**, 1–6 (2020).

149. J. L. Doman, A. Raguram, G. A. Newby, D. R. Liu, Evaluation and minimization of Cas9-independent off-target DNA editing by cytosine base editors. *Nat. Biotechnol.* **38**, 620–628 (2020).

150. D. Reichart, G. A. . Newby, H. Wakimoto, M. Lun, J. M. Gorham, J. J. Curran, A. Raguram, D. M. DeLaughter, D. A. Conner, J. D. C. Marsiglia, S. Kohli, L. Chmatal, D. C. Page, N. Zabaleta, L. Vandenberghe, D. R. Liu, J. G. Seidman1, C. Seidman, Efficient in vivo Genome Editing Prevents Hypertrophic Cardiomyopathy in Mice. *Nat. Med.* **in press** (2022).

151. X. Huang, H. Guo, S. Tammana, Y. C. Jung, E. Mellgren, P. Bassi, Q. Cao, Z. J. Tu, Y. C. Kim, S. C. Ekker, X. Wu, S. M. Wang, X. Zhou, Gene transfer efficiency and genome-wide integration profiling of sleeping beauty, Tol2, and PiggyBac transposons in human primary t cells. *Mol. Ther.* **18**, 1803–1813 (2010).

152. J. A. Briggs, V. C. Li, S. Lee, C. J. Woolf, A. Klein, M. W. Kirschner, Mouse embryonic stem cells can differentiate via multiple paths to the same state. *Elife*. **6**, e26945 (2017).

153. S. M. Ryu, T. Koo, K. Kim, K. Lim, G. Baek, S. T. Kim, H. S. Kim, D. E. Kim, H. Lee, E. Chung, J. S. Kim, Adenine base editing in mouse embryos and an adult mouse model of Duchenne muscular dystrophy. *Nat. Biotechnol.* **36**, 536–539 (2018).

154. J. Zettler, V. Schütz, H. D. Mootz, The naturally split Npu DnaE intein exhibits an extraordinarily high rate in the protein trans-splicing reaction. *FEBS Lett.* **583**, 909–914 (2009).

155. C. K. W. Lim, M. Gapinske, A. K. Brooks, W. S. Woods, J. E. Powell, M. A. Zeballos C., J. Winter, P. Perez-Pinera, T. Gaj, Treatment of a Mouse Model of ALS by In Vivo Base Editing. *Mol. Ther.* (2020), doi:10.1016/j.ymthe.2020.01.005.

156. S. Banskota, A. Raguram, S. Suh, S. W. Du, J. R. Davis, E. H. Choi, X. Wang, S. C. Nielsen, G. A. Newby, P. B. Randolph, M. J. Osborn, K. Musunuru, K. Palczewski, D. R. Liu, Engineered virus-like particles for efficient in vivo delivery of therapeutic proteins. *Cell*. **185**, 250-265.e16 (2022).

157. C. M. Lutz, S. Kariya, S. Patruni, M. A. Osborne, D. Liu, C. E. Henderson, D. K. Li, L. Pellizzoni, J. Rojas, D. M. Valenzuela, A. J. Murphy, M. L. Winberg, U. R. Monani, Postsymptomatic restoration of SMN rescues the disease phenotype in a mouse model of severe spinal muscular atrophy. *J. Clin. Invest.* **121**, 3029–3041 (2011).

158. S. Paushkin, A. K. Gubitz, S. Massenet, G. Dreyfuss, The SMN complex, an assemblyosome of ribonucleoproteins. *Curr. Opin. Cell Biol.* **14**, 305–312 (2002).

159. A. K. Gubitz, W. Feng, G. Dreyfuss, The SMN complex. *Exp. Cell Res.* **296**, 51–56 (2004).

160. Y. B. Chan, I. Miguel-Aliaga, C. Franks, N. Thomas, B. Trülzsch, D. B. Sattelle, K. E. Davies, M. van den Heuvel, Neuromuscular defects in a Drosophila survival motor neuron gene mutant. *Hum. Mol. Genet.* **12**, 1367–1376 (2003).

161. E. Szunyogova, H. Zhou, G. K. Maxwell, R. A. Powis, M. Francesco, T. H. Gillingwater, S. H. Parson, Survival Motor Neuron (SMN) protein is required for normal mouse liver development. *Sci. Rep.* **6** (2016), doi:10.1038/srep34635.

162. H. Chaytow, Y. T. Huang, T. H. Gillingwater, K. M. E. Faller, The role of survival motor neuron protein (SMN) in protein homeostasis. *Cell. Mol. Life Sci.* **75** (2018), pp. 3877–3894.

163. A. J. Blatnik, V. L. McGovern, T. T. Le, C. C. Iyer, B. K. Kaspar, A. H. M. Burghes, Conditional deletion of SMN in cell culture identifies functional SMN alleles. *Hum. Mol. Genet.* **29**, 3477–3492 (2020).

164. S. Sivanesan, M. D. Howell, C. J. Didonato, R. N. Singh, Antisense oligonucleotide mediated therapy of spinal muscular atrophy. *Transl. Neurosci.* **4** (2013), pp. 1–7.

**Video S1. (separate file)**

**Untreated Δ7SMA mice at PND14.**

**Video S2. (separate file)**

**AAV9-ABE-treated Δ7SMA mice at PND14.**

**Video S3. (separate file)**

**PND40 voluntary movement of AAV9-ABE+nusinersen-treated Δ7SMA mice**.

Shows an AAV9-ABE+nusinersen-treated Δ7SMA mouse (left) and a healthy heterozygous littermate (right), both at PND40.

**Video S4. (separate file)**

**PND96 voluntary movement of AAV9-ABE+nusinersen-treated Δ7SMA mice.**

Shows an AAV9-ABE+nusinersen treated Δ7SMA mouse (below) and a healthy heterozygous littermate (above), both at PND96.

**Video S5. (separate file)**

**PND200 voluntary movement of AAV9-ABE+nusinersen-treated Δ7SMA mice.**

Shows an AAV9-ABE+nusinersen treated Δ7SMA mouse (below) and a healthy heterozygous littermate (above), both at PND200.
